# Analysis of fecal bile acids and metabolites by high resolution mass spectrometry in farm animals and correlation with microbiota

**DOI:** 10.1038/s41598-022-06692-9

**Published:** 2022-02-21

**Authors:** Emanuele Porru, Daniel Scicchitano, Nicolò Interino, Teresa Tavella, Marco Candela, Aldo Roda, Jessica Fiori

**Affiliations:** 1 Department of Chemistry “G. Ciamician”, University of Bologna, via Selmi 2, 40126 Bologna, Italy; 2 Department of Pharmacy and Biotechnology, University of Bologna, via Belmeloro 6, 40126 Bologna, Italy; 3 INBB-Biostructures and Biosystems National Institute, viale delle Medaglie d’Oro 305, 00136 Rome, Italy; 4 Interdepartmental Center for Industrial Research–CIRI-MAM, University of Bologna, Bologna, Italy

**Keywords:** Biochemistry, Chemical biology, Microbiology, Biomarkers, Chemistry

## Abstract

There is a growing interest in the named “acidic sterolbiome” and in the genetic potential of the gut microbiome (GM) to modify bile acid (BA) structure. Indeed, the qualitative composition of BAs in feces correlates with the bowel microorganisms and their collective genetic material. GM is responsible for the production of BA metabolites, such as secondary and oxo-BAs. The specific BA profiles, as microbiome-host co-metabolic products, could be useful to investigate the GM-host interaction in animals under physiological conditions, as well as in specific diseases. In this context, we developed and validated an ultra-performance liquid chromatography-quadrupole time-of-flight mass spectrometry method for the simultaneous analysis of up to 21 oxo-BAs and their 9 metabolic precursors. Chromatographic separation was achieved in 7 min with adequate analytical performance in terms of selectivity, sensitivity (LOQ from 0.05 to 0.1 µg/mL), accuracy (bias% < 5%), precision (CV% < 5%) and matrix effect (ME% < 10%). A fast solvent extraction protocol has been fine-tuned, achieving recoveries > 90%. In parallel, the gut microbiota assessment in farming animals was evaluated by 16S rRNA next-generation sequencing, and the correlation with the BA composition was performed by multivariate analysis, allowing to reconstruct species-specific associations between the BA profile and specific GM components.

## Introduction

Bile acids (BAs) are the end products of cholesterol catabolism in more than 650 vertebrate species^1^. They are synthetized by the liver and once conjugated with glycine/taurine secreted in bile into the proximal intestine. A major portion of conjugated BAs, is selectively and efficiently actively absorbed in the terminal ileum; the unconjugated fraction is passively absorbed along the entire intestinal tract. All the absorbed BAs reach the liver via the portal vein, where they are taken up by the liver and partial spillover in the systemic blood take place. Therefore, BAs are recycled in the enterohepatic circulation, where they exert their physiological function in bile to facilitate lipid absorption via mixed micelles thanks to their detergency. The dynamic of their recycling is driven by the gallbladder (if present) and intestinal motility^2^. In addition, as already demonstrated, they are ligands of nuclear receptors, such as farnesoid X receptor (FXR), largely expressed in the liver and intestine, which is involved in several biological pathways, including BA own synthesis. The finding of their “hormone activity” at the end of the twentieth century has elicited an increasing interest in the BAs family, especially for their potential use as drugs in several hepato-biliary diseases. For example, semisynthetic Obeticholic acid is recognized as the most potent (FXR) agonist and is used as a drug to treat primary biliary cholangitis, and it is undergoing development for deployment in further liver diseases and related disorders^2^, such as nonalcoholic steatohepatitis (NASH).

No other class of small molecules in vertebrates exhibits the variety of chemical structures shown by BAs, and the identification of new bile alcohols and BA derivatives continues to date^3^. BA chemical structures vary in three main ways: (a) structure of the side chain, which determines the BA class; (b) stereochemistry of the A/B juncture [cis (5b) or trans (5a)]; and (c) hydroxylation pattern (number, position and orientation) of the nucleus and/or side chain.

This huge chemical diversity is also related to the fact that the ligand binding pocket of the BA receptors has changed across species during the evolution process of vertebrates^4^. For example, human FXR has a curved binding pocket best suited for the bent steroid ring structure typical of evolutionarily more recent BAs. Several isoforms of FXR have been determined in mice and are differenty expressed in liver, intestine, kidney, adrenal gland, stomach, fat, and heart, where they differentially regulate gene expression in numerous tissues in vivo^5^.

Each vertebrate therefore presents a different pattern of cholesterol metabolites, as the result of evolutionary changes^6^. Several papers have reported the BAs composition in numerous animals, specifically in bile and blood samples^7^. The study of BA variations across vertebrates, has highlighted a clear evidence of the evolutionary transition from C27 ancestral bile alcohols to C24 BAs, with an intermediate phase in some animal classes where C27 BAs are present.

The complexity of the BAs family, named the “acidic steroid profile”, is also due to the interaction with the intestinal microbiome and the resulting microbiome-host co-metabolic network with species-specific declinations. Indeed, it is well known that a small percentage of BAs not absorbed by the intestine across the ileal-caecum valve, enters the colon, where in the presence of the bacterial microflora is extensively metabolized. Bacteria bile salt Hydrolase (BSH) is the main metabolic pathway in gut which is responsible for the cleavage of the amide bond with glycine and /or taurine to produce unconjugated BAs. Metabolites are partially reabsorbed by the colon, and the remaining metabolites are excreted in the stools. The amount of excreted BAs is balanced by the BAs of nova synthesis from cholesterol in the liver by a feedback mechanism, thus ensuring the steady-state BA pool size and acting as a way to control cholesterol excretion.

In this process, since different host species possess a species-specific pattern of gut microbial components with an interspecies gut microbiome diversity matching the host phylogenetic distance, the species-specific gut microbiome structural and functional profile combines with the host genome in the definition of the “acidic sterolbiome” of a peculiar vertebrate host^9^. In particular, different strains of bacteria can metabolize steroid compounds during their intestinal transit, leading to the formation of a number of BAs derivatives that can therefore have different activities towards receptors as the major transcriptional regulator of BAs synthesis FXR^8^. Indeed, the bacteriostatic effect of BAs on the colon content is known, but the specific BAs structure action is not yet well defined, and the bacterial specificity is still poorly understood.

**Figure 1:**
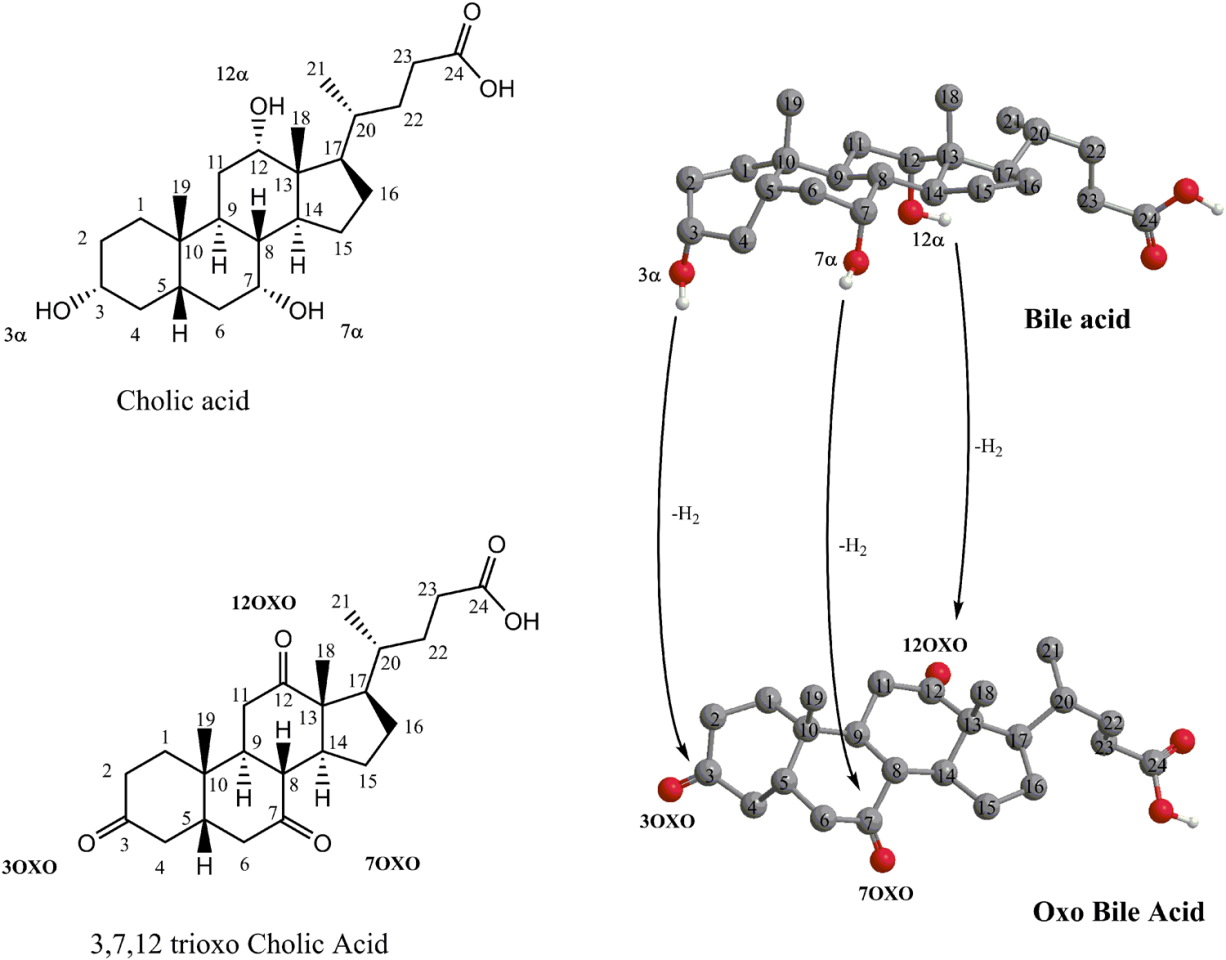
Cholic acid and one of its potential metabolic derivatives, (5β)-3,7,12-trioxocholan-24-oic acid, are produced by microbiota dehydrogenase.

Among gut microbiome metabolites, keto (or oxo) BAs produced by steroid dehydrogenase enzyme expressed by microbiota, mainly 3,7 and 12 Hydroxysteroid dehydrogenases (HSD), have been onlyanalyzed in human faeces^9^. The cholic acid (CA) structure and one of its potential metabolic derivatives, (5β)-3,7,12-trioxocholan-24-oic acid, are reported in Fig. 1.

There are low concentrations of these species in human blood due to the reduction step of the oxo groups by liver after their hepatic uptake. On the other hand, high levels of these molecules in the entire intestinal tract have been determined. Moreover, high fecal concentrations of several oxo bile acids (oxo-BAs) have been highlighted by HPLC–ESI–MS methods, specifically oxo-derivatives of the secondary BA deoxycholic acid (DCA). Indeed, such hixi-derivatives cover a significant fraction of fecal BAs, approximately 30% of the total pool^9,10^.

These oxo BA derivatives have never been properly evaluated in either human or animal feces. The lack of information about oxo-BAs in vertebrate feces hampers the evaluation of their physiological role, for example their activity as endogenous ligands for receptors or as bacteriostatic agents^11^. In addition, considering their nature as microbiome-host co-metabolic products, more detailed knowledge of the fecal acidic steroid profile could represent a valuable tool to study gut microbiota-host interactions in vertebrates under physiological and pathological conditions^12^, as well as to establish fingerprinting biomarkers useful for feces characterization (e.g. in archaeological studies on coprolites). In fact, thanks to their high stability and specificity, BAs and oxo-BA metabolites are promising biomarkers for the identification of human fecal inputs, giving a significant contribution to the growing field of bioarchaeology^10^.

High resolution mass spectrometry is definitely the most powerful and comprehensive technique to investigate metabolic profiles due to its extreme selectivity. The coupling of mass spectrometry with chromatographic techniques of the latest generation has allowed in recent years to study and identify new metabolic pathways, and their products, on molecules of endogenous and exogenous origin^13,14^. Here, we aimed to investigate the BAs profile mainly in livestock animal feces to develop new tools potentially applicable to future studies on farm animal welfare, based on the evidence that BAs are powerful species-specific markers related to microbiota and disease conditions in these species. For example, the identification of negative effects of intensive farming and specific feeds, as well as displacement of physiological condition in animals, could be better achieved with higher capillarity. Indeed, several papers suggest controversial or poor specific effects on the BA profile by diet in vertebrates^8^, but oxo-BAs and their strict interaction with the host gut microbiota have been actually overlooked so far.

The analysis of BAs in plasma, bile and, more recently, in fecal samples has been extensively reported and discussed in literature^15^. The analysis of BAs in biological liquids requires the use of highly selective analytical techniques, due to the complexity of either their profile and the sample matrix. Indeed, due to the multiplicity of the BAs structures (Fig. 1) in the feces (i.e., primary and secondary BAs, oxo-BAs, epimers) and the relatively high range of polarities, long analysis times often still unable to detect the multiplicity of BAs and GM metabolites, are required.

With the aim to improve the analytical capabilities required to fully characterize the acidic sterolbiome of farm animals, we developed and validated a comprehensive and fast ultra-performance liquid chromatography-quadrupole time-of-flight mass spectrometry (UPLC-QTOF-MS) method for the analysis of 30 compounds, 21 oxo-BAs and their metabolic precursors, in 10 vertebrates. This UPLC method permits faster identification and quantification with high selectivity thanks to the use of new-generation high-resolution mass spectrometry (HR-MS). The UPLC-MS method was fully validated to achieve high analytical performances in terms of reproducibility, accuracy, selectivity, and sensitivity in fecal samples.

The extraction procedure and percentage BAs recovery were evaluated for each animal. This step was necessary on the basis of the great variability of diets (herbivores and omnivores) and digestive systems^16^, which is reflected in the overall bulk composition of the stool samples. Furthermore, a systematic evaluation of the MS/MS spectra was carried out to identify characteristic fragmentation patterns. Specific fragments and their different relative abundances can in fact be diriment in the discrimination of isobaric compounds with similar chromatographic behaviour.

Additionally, the present study deals with gut microbiota assessment in farming animals wvia 16S rRNA next-generation sequencing, which successfully allowed to reconstruct species-specific associations between the BA profile and specific gut microbiome components.

## Material and methods

### Chemicals

Methyl alcohol, isopropyl alcohol, and acetonitrile (ACN), hypergrade for LC–MS (Lichrosolv), were purchased from Sigma-Aldrich (St. Louis, Missouri, United States). Acetic acid (98% pure), and ammonium hydroxide (98% pure) were purchased from Fluka (Buchs, Switzerland). Water of HPLC–MS grade (Millipore) was produced by Milli-Q Synthesis A 10 (Molsheim, France). Authentic chemical standards of the BAs and deuterated BAs (all provided as sodium salts) were purchased from Sigma-Aldrich (Saint Louis, USA). Commercially available standards of the oxo-BAs were purchased from Steraloids (Newport, USA).

Standards of the commercially unavailable compounds were synthesized following a procedure previously reported^9^. The purity of the synthetized compounds was determined by NMR and HPLC coupled with high-resolution mass spectrometry. All the standards used in this study were > 95% pure (Table 1). Our abbreviations for oxo-BAs reflect the structure of the respective metabolic precursor, indicating the position involved in the oxidative reaction (i.e., ‘3oxo LCA’ means LCA with a hydroxy group converted to an oxo group at position 3 of the steroid ring).

**Table 1:** List of the analysed BAs and their metabolites with abbreviations, retention times, and quantifier/qualifier ions.

	Retention time (min)	Quantifier/qualifier (mz^−1^)
**Oxo-BA (abbreviation)**		
3,7,12-trioxo-5﻿β-cholan-24-oic acid (trioxo-CA)	2.01	[401.2302]–[401.2302]
7α,12β-dihydroxy-3-oxo-5﻿β-cholan-24-oic acid (12β-3-oxo-CA)	2.39	[405.2699]–[359.2630]
7β,12α-dihydroxy-3-oxo-5﻿β-cholan-24-oic acid (3-oxo-UCA)	2.57	[405.2699]–[271.2118]
3α,6α-dihydroxy-7-oxo-5﻿β-cholan-24-oic acid (7-oxo-HCA)	3.28	[405.2699]–[405.2699]
3α,12α-dihydroxy-7-oxo-5﻿β-cholan-24-oic acid (7-oxo-CA)	3.36	[405.2699]–[289.2219]
3α,7α-dihydroxy-12-oxo-5﻿β-cholan-24-oic acid (12oxo-CA)	3.47	[405.2699]–[245.1555]
3,7-dioxo-5﻿β-cholan-24-oic acid (3,7-dioxo-CDCA)	3.67	[387.2588]–[273.2253]
6α,7α-dihydroxy-3-oxo-5﻿β-cholan-24-oic acid (3-oxo-HCA)	3.62	[405.2699]–[385.2466]
3,12-dioxo-5﻿β-cholan-24-oic acid (3,12-dioxo-DCA)	3.78	[387.2588]–[341.2532]
7β-hydroxy-3-oxo-5﻿β-cholan-24-oic acid (3-oxo-UDCA)	3.77	[389.2762]–[389.2762]
3,6-dioxo-5﻿β-cholan-24-oic acid (3,6-dioxo-HDCA)	3.85	[387.2588]–[309.2280]
7α,12α-dihydroxy-3-oxo-5β-cholan-24-oic acid (3-oxo-CA)	3.95	[405.2699]–[289.2219]
6α-hydroxy-3-oxo-5﻿β-cholan-24-oic acid (3-oxo-HDCA)	3.88	[389.2762]–[389.2762]
3α-hydroxy-6-oxo-5﻿β-cholan-24-oic acid (6-oxo-HDCA)	3.95	[389.2762]–[389.2762]
3α-hydroxy-7-oxo-5﻿β-cholan-24-oic acid (7-oxo-CDCA)	4.11	[389.2762]–[389.2762]
12β-hydroxy-3-oxo-5﻿β-cholan-24-oic acid (12β-3-oxo-DCA)	4.21	[389.2762]–[343.2685]
3α-hydroxy-12-oxo-5﻿β-cholan-24-oic acid (12-oxo-DCA)	4.27	[389.2762]–[343.2685]
3α-hydroxy-6,7-dioxo-5﻿β-cholan-24-oic acid (6,7-dioxo-CA)	3.87	[403.2380]–[403.2380]
7α-hydroxy-3-oxo-5﻿β-cholan-24-oic acid (3oxo-CDCA)	4.63	[389.2762]–[389.2762]
12α-hydroxy-3-oxo-5﻿β-cholan-24-oic acid (3oxo-DCA)	4.74	[389.2762]–[389.2762]
3-oxo-5﻿β-cholan-24-oic acid (3oxo-LCA)	5.17	[373.2741]–[373.2741]
**BA (abbreviation)**		
3α,6β,7α-trihydroxy-5﻿β-cholan-24-oic acid (αMCA)	3.23	[407.2840]–[387.2507]
3α,6β,7β-trihydroxy-5﻿β-cholan-24-oic acid (βMCA)	3.32	[407.2840]–[371.2571]
3α,6α,7α-trihydroxy-5﻿β-cholan-24-oic acid (HCA)	3.93	[407.2840]–[389.2721]
3α,7β-dihydroxy-5﻿β-cholan-24-oic acid (UDCA)	3.98	[391.2906]–[373.2760]
3α,6α-dihydroxy-5﻿β-cholan-24-oic acid (HDCA)	4.15	[391.2906]–[391.2760]
3α,7α,12α-trihydroxy-5﻿β-cholan-24-oic acid (CA)	4.42	[407.2840]–[289.2184]
3α,7α-dihydroxy-5﻿β-cholan-24-oic acid (CDCA)	4.96	[391.2906]–[373.2799]
3α,12α-dihydroxy-5﻿β-cholan-24-oic acid (DCA)	5.07	[391.2906]–[343.2886]
3α-hydroxy-5﻿β-cholan-24-oic acid (LCA)	5.58	[375.3002]–[355.2726]

Isopropyl alcohol, methyl alcohol (CH3OH), and acetonitrile (ACN), all of MS grade, were purchased from Merck (Darmstadt, Germany). Acetic acid, formic acid, and ammonium hydroxide (all 98% pure) were purchased from Fluka (Buchs, Switzerland). Milli-Q Synthesis A 10 depurative system (Molsheim, France) was used to produce water of high purity grade. Other solvents were all analytical grade.

Stock solutions of each analyte and IS were prepared in isopropanol at a concentration of 1 mg/mL and stored at − 20 °C as the procedure already reported^9^. These stock solutions were further diluted in isopropanol to obtain working solutions, stored at 4 °C and used for 4 weeks at most.

### Animals and humans

Fecal samples were collected from 9 different mammalian vertebrates and one nonmammalian vertebrate: pig, wild boar, dog, rabbit, mouse, chicken, horse, donkey and cow. Ten healthy adult subjects, 5 males and 5 females, were selected for all investigated animals, except for wild boar samples that were collected without sex checks. The farm animals were under a regular diet from antibiotic-free and not intensively exploited livestock. The study was carried out in accordance with ARRVE guidelines. Ethical review and approval was not required for the animal study because faces were collected from hosted animals in farms without touching nor disturbing them in any way.

Fecal samples were collected also from 10 healthy human subjects (experimental protocol was approved by Ethics Committee of St. Orsola Hospital protocol No. 7-2209-USPER), 5 males and 5 females, from 18 and 60 years old, with no colorectal disorders, digestive, biliary and other gastrohepatic/hepatogastric disorders. Informed consent was obtained from all subjects and/or their legal guardian(s), and all methods were carried out in accordance with the Declaration of Helsinki guidelines and regulations.

### Sample treatment

The fecal extraction has been optimized according to the different procedures for the most common BAs reported in the literature^15^. Four different procedures were tested for oxo-BAs in animals: isopropanol, acetonitrile, methanol extractions and sodium hydroxide 0.1 M.

Samples were obtained fresh and kept on ice until storage at − 80°. According to our results for oxo-BA extraction, sample preparation was performed following the selected conditions described below, already reported in our paper with minor modification^9^. Aliquots of wet fecal sample homogenate (1 g) were extracted with 3 mL of isopropyl alcohol. The mixture was stirred for 30 min at 37 °C and then centrifuged at 600 g for 10 min. The supernatant was then diluted 1:10 (v/v) with 40% isopropanol in 15 mM ammonium acetate at pH 8.00, filtered, transferred to an autosampler vial, and 1 μL injected into the UPLC-QTOF-MS system. The results obtained from the analysis are expressed as μg/g of wet feces.

### UPLC-HR-MS analytical method

The choice of the chromatographic and mass spectrometry conditions were based on our previous HPLC–MS/MS method for BAs^9^. Liquid chromatography was performed using an ACQUITY UPLC (Waters, Milford, MA, USA) Waters BEH C18 column (100 mm × 2 mm, 1.7 μm) kept at 37 °C throughout the analyses. The mobile phase was composed of 15 mM ammonium acetate buffer at pH 8.00 (phase A) and methanol 99.92% pure (phase B). Final separation was achieved at a 0.3 mL/min flow rate under gradient elution conditions: 40% B for 0.1 min, 40–55% B from 0.1 to 0.4 min, 55% from 0.4 to 1 min, 55–65% B from 1 to 2.1 min, 65–80% B from 2.1 to 3.3 min, and 90% B from 3.3 to 7 min. Re-equilibration at 40% B between analyses was achieved in 4 min for a total run time of 11 min. The injected sample volume was 1 μL. The autosampler temperature was kept at 10 °C to preserve the sample from precipitation.

Mass spectrometry analysis was performed using a Qtof system (Waters, Xevo G2-XS) with an ESI source. High-resolution multiple reaction monitoring acquisition mode (HR-MRM) was used for quantification.

The major fragment for quantification and the qualifier ion fragments for identification (Table 1) were determined from injection of a solution of each analyte at 5 µg/mL in the mobile phase. The deprotonated molecular ions were used as quantifiers due to the stability of these molecules in tandem mass spectrometry using high collision energy.

The detection parameters of the ESI source were as follows: capillary voltage 2.5 kV; sample cone 40 V; source offset 80 V; source temperature 100 °C; declustering potential 80 eV; flow rate of cone gas 50 L/h; temperatures and flow rate of desolvation gas (N_2_) 400 °C and 800 L/h, respectively; and collision energy of 40 eV were the same for all compounds. The software UNIFI was used to control the instrument and acquire and analyse data.

### Validation of the UPLC-HR-MRM method

The linearity of the method was verified by plotting the peak area ratio of each compound to IS versus their concentrations. Each calibration curve was performed with six appropriate concentrations (0.05, 0.1, 0.5, 1, 5, 10, and 20 μg/mL), obtained by diluting the working solution with mobile phase and analysed in duplicate. Internal standard (IS) concentrations were kept constant at 1 µg/mL for LCA-D4, CA-D4 and DCA-D4. According to the similar chemical structures and to obtain the best available IS for each analyte, CA-D4 was used to quantify CA, MCA and oxo-CAs; DCA-D4 was used to quantify secondary BAs and their oxo metabolites; LCA-D4 was used to quantify LCA and 3-OXO LCA.

The limit of detection (LOD, S/N = 3) and limit of quantification (LOQ, S/N = 10) of the method for each single analyte were experimentally determined by injecting stepwise dilutions of the standard solution. Accuracy (bias%) and precision (CV%) were determined intra- and inter daily through triplicate analysis over three different days (n = 9) by analysing mixed standard solutions at low (0.2 μg/mL), medium (2 μg/mL), and high (15 μg/mL) concentration levels.

The matrix effect was evaluated by comparing standard calibration curve slopes and matrix-matched calibration curve slopes with a t-test (*p* > 0.05).

The fecal BA and oxo-BA extraction procedure was optimized and validated. Four different solvents were tested: isopropanol as described in paragraph 2.1; acetonitrile and methanol with the same procedure adopted for the isopropyl alcohol, and sodium hydroxide 0.1 M followed by C18 solid phase extraction. For sodium hydroxide extraction, 5 mL of NaOH (0.1 mol/L) was added to 1 g of feces and incubated for 1 h at 60 °C before the addition of 4 mL of water. The sample was homogenized and centrifuged at 600 g for 10 min. The supernatant was collected and extracted using a 500 mg SPE cartridge C18. Preconditioning was carried out with 5 mL methanol and 5 mL water. The cartridge was loaded and rinsed successively with 10 mL water to discard salts and hydrophilic metabolites. BAs were then eluted with 5 mL methanol. The methanol fraction was collected and dried under a nitrogen stream. The residue was dissolved in 150 µL methanol, and 1 µL was injected into the mass spectrometer.

Recovery was assessed for each extraction protocol in each animal by comparing the areas of standard fecal extracts (2 µg/mL) fortified before extraction with those fortified after extraction. The results are obtained from 3 different experiment at least.

According to our results, the recovery for the best extraction protocol (isopropyl alcohol) was also evaluated for each analyte in human fecal sample at three concentration levels (0.2 μg/mL, 2 μg/mL and 15 μg/mL).

### Microbiota analysis

Total bacterial DNA was extracted from individual fecal samples obtained from the following animals: pig, wild boar, dog, rabbit, chicken, horse, donkey, using the DNeasy Blood & Tissue Kit (QIAGEN, Hilden, Germany) with a modified protocol as previously shown^17^. Briefly, the homogenization step was performed three times using the FastPrep instrument (MP Biomedicals, Irvine, CA) at 5.5 movements per sec for 1 min. Afterward, samples were heated at 95 °C for 15 min. Then, at the end of the purification step, DNA was quantified by using a NanoDrop ND-1000 (NanoDrop Technologies, Wilmington, DE) and stored at − 20 °C until PCR amplification and library preparation. After the isolation of microbial DNA from fecal samples, the amplification of the V3–V4 hypervariable region of the 16S rRNA gene was carried out using the 341F and 785R primers^18^ with added Illumina adapter overhang sequences, as previously reported^19^. PCRs were purified by using Agencourt AMPure XP magnetic beads (Bckman Coulter, Brea, CA), and Nextera Technology was used to prepare indexed libraries by limited-cycle PCR. After a further clean-up step as described above, libraries were normalized to 4 nM and pooled. Sequencing was performed on an Illumina MiSeq platform using a 2 × 250 bp paired end protocol, according to the manufacturer's instructions (Illumina, San Diego, CA).

### Statistics and bioinformatics

Data analysis for BAs quantification was performed using GraphPad Prism 5.0 software (La Jolla, CA, USA). The quantitative data are presented as the mean (µg/g_wet feces_) ± standard deviation (SD) and as a percentage with respect to the total quantity of BAs.

Raw microbial sequences were processed using a pipeline combining PANDASEQ^20^ and QIIME^21^ as previously reported in Biagi et al.^17^. High-quality reads, obtained by a filtering step for length and quality with default parameters in QIIME, were binned into operational taxonomic units (OTUs) according to a 99% similarity threshold using UCLUST^22^. Taxonomy was assigned using the RDP classifier against the SILVA database^23^. OTUs were filtered for their presence in at least 5 samples.

Alpha rarefactions were analyzed by using Faith's phylogenetic diversity (PD whole tree), OTU species count (observed species) and Chao1 index metrics for microbial richness.

All gut microbiota statistical analyses were performed using the R project (https://www.r-project.org/). Beta diversity was estimated by computing unweighted UniFrac distance, which was used as input for principal coordinates analysis (PCoA) to explore inter-sample variability in relation to the different animal species. The contribution of covariates to the ordination space was found by using the function ‘envfit’ of the R package vegan. A permutation test with pseudo-F ratios (function ‘adonis’ in the vegan package) was used to determine the significance of separation on the PCoA plot. The Kruskal–Wallis test followed by the post hoc Wilcoxon rank-sum test were used to assess significant differences in taxon/BA relative abundance between animal groups. The hierarchical cluster analysis was computed on the matrices of relative abundances (samples-genera, samples-bile acids) with the function ‘hclust’, using as distance the Pearson correlation coefficient, and Ward’s linkage method (ward. D2 function). The optimal number of clusters was defined with the elbow method using the function ‘fviz_nbclust’ in the factoextra package. Correlation analysis was performed using the ‘rcorr’ function in R with the Pearson method, which allowed us to find any correlation (positive or negative) between BAs and bacterial genera. Univariate linear regressions were computed with the function ‘lm’ in R. P-values were adjusted using the false discovery rate (FDR) function in R, and a *p* value ≤ 0.05 was considered statistically significant.

## Results

### Bile acids UPLC-HR-MS analysis

**Figure 2:**
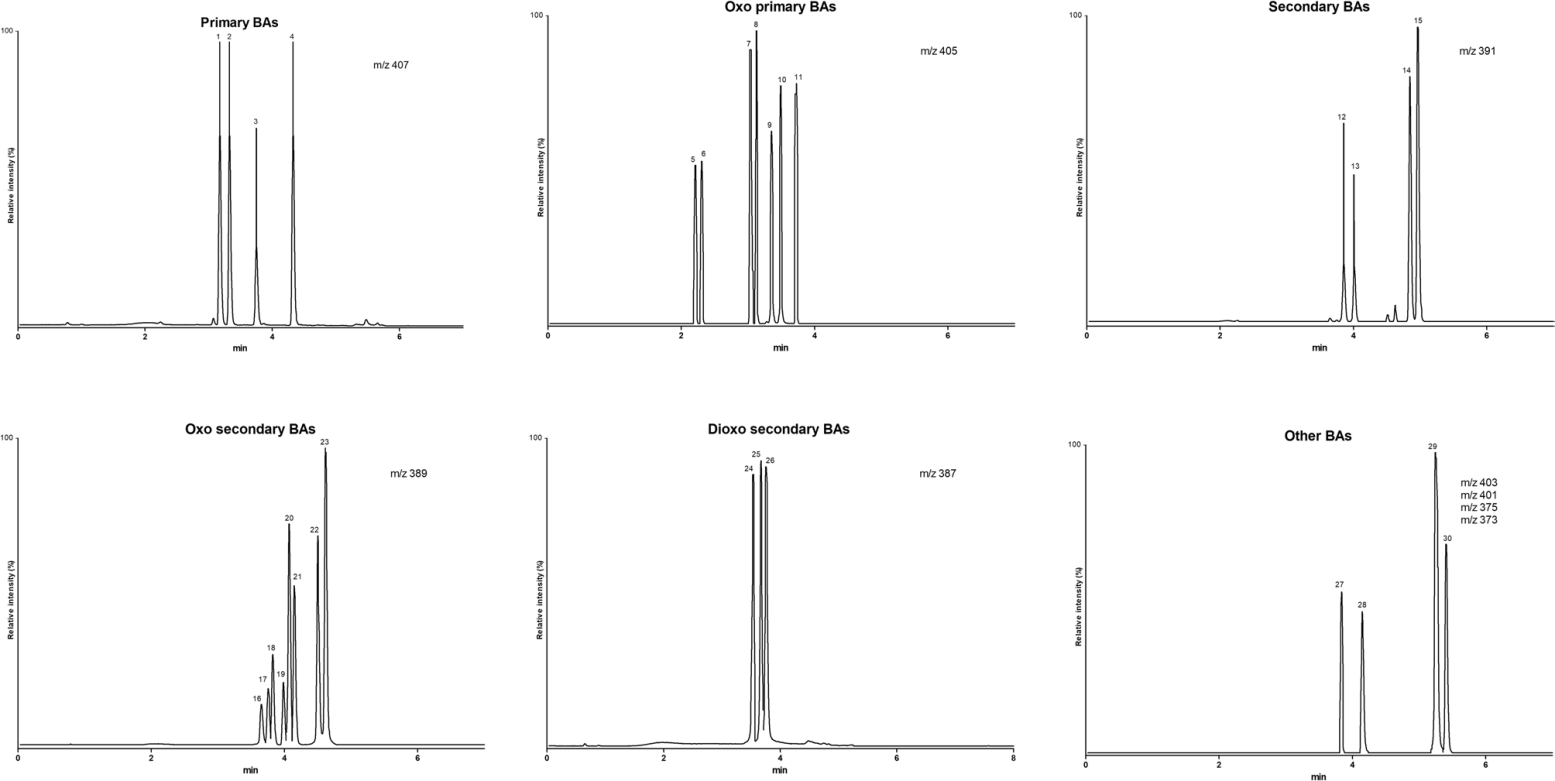
Chromatograms of the investigated BAs and oxo derivatives. 3α,6β,7α-trihydroxy-5β-cholan-24-oic acid (1), 3α,6β,7β-trihydroxy-5β-cholan-24-oic acid (2), 3α,6α,7α-trihydroxy-5β-cholan-24-oic acid (3), 3α,7α,12α-trihydroxy-5β-cholan-24-oic acid (4), 7α,12β-dihydroxy-3-oxo-5β-cholan-24-oic acid (5), 7β,12α-dihydroxy-3-oxo-5β-cholan-24-oic acid (6), 3α,6α-dihydroxy-7-oxo-5β-cholan-24-oic acid (7), 3α,12α-dihydroxy-7-oxo-5β-cholan-24-oic acid (8), 3α,7α-dihydroxy-12-oxo-5β-cholan-24-oic acid (9), 6α,7α-dihydroxy-3-oxo-5β-cholan-24-oic acid (10), 7α,12α-dihydroxy-3-oxo-5β-cholan-24-oic acid (11), 3α,7β-dihydroxy-5β-cholan-24-oic acid (12), 3α,6α-dihydroxy-5β-cholan-24-oic acid (13), 3α,7α-dihydroxy-5β-cholan-24-oic acid (14), 3α,12α-dihydroxy-5β-cholan-24-oic acid (15), 7β-hydroxy-3-oxo-5β-cholan-24-oic acid (16), 6α-hydroxy-3-oxo-5β-cholan-24-oic acid (17), 3α-hydroxy-6-oxo-5β-cholan-24-oic acid (18), 3α-hydroxy-7-oxo-5β-cholan-24-oic acid (19), 12β-hydroxy-3-oxo-5β-cholan-24-oic acid (20), 3α-hydroxy-12-oxo-5β-cholan-24-oic acid (21), 7α-hydroxy-3-oxo-5β-cholan-24-oic acid (22), 12α-hydroxy-3-oxo-5β-cholan-24-oic acid (23), 3,7-dioxo-5β-cholan-24-oic acid (24), 3,12-dioxo-5β-cholan-24-oic acid (25), 3,6-dioxo-5β-cholan-24-oic acid (26), 3,7,12-trioxo-5β-cholan-24-oic acid (27), 3α-hydroxy-6,7-dioxo-5β-cholan-24-oic acid (28), 3-oxo-5β-cholan-24-oic acid (29), 3α-hydroxy-5β-cholan-24-oic acid (30).

The retention times of each BA were between 1 and 7 min under the chromatographic conditions described above, resulting in adequate resolution for all the analysed molecules. Representative ion chromatograms of the deprotonated molecules [M–H]^−^ are shown in Fig. 2.

The choice of the mobile phase was based on our previous HPLC–MS/MS method for oxo-BAs^9^. The elution gradient was modified to obtain the best chromatographic separation, avoiding the overlap of structural isomers. The best temperature to optimize the retention times and peak symmetry was 37 °C.

The oxo-BAs were first detected by UPLC-QTOF-MS/MS in both positive and negative ionization modes. The sensitivity obtained from the negative ion mode was tenfold higher than that obtained from the positive ion mode. Thus, the ESI^−^ mode was selected for quali-quantitative analysis of the compounds. Single oxo-BA TOF–MS scans were set at their m/z value followed by an MS/MS product ion scan from 70 to 500 Da. No product ions were used for quantification because better sensitivity (higher signal-to-noise ratio) was experienced working in HR-MRM mode monitoring the transition [M–H]^−^  > [M–H]^−^, pseudo-MRM, while the other product ions were used for the qualitative analysis of the samples (Table 1).

### MS/MS fragmentation pattern analysis of bile acids and oxo bile acids

Free oxo-BAs and free BAs exhibited abundant [M–H]^−^ ions even at high collision energy. However, solvent adduction and neutral loss patterns of the isomeric BAs varied remarkably and could be used for their differentiation.

A more complex fragmentation pattern was detected in the spectra of BAs possessing a 12-hydroxyl group^24^. The ion [M–H–H_2_O–CH_2_O_2_]^−^ (343.3 m/z) is one of the major fragments for CA, along with the fragment ion [C_19_H_29_O_2_]^−^ (289.2 m/z) (Supplementary material Fig. S1). The fragmentation of CA is characterized by a more distinct cleavage pattern not observed for secondary BAs, resulting in a series of product ions within the m/z range of 180–300. According to Lan et al.^25^, they are associated with the cleavage pattern of the steroid skeleton of ring-A, from C1–C10 to C3–C4 [C_19_H_29_O_2_]—(289.2 m/z), from the cleavages of ring-B, from C5–C6 to C9–C10 [C_16_H_27_O_2_]—(251.20 m/z), and from C6–C7 to C9–C10 cleavage [C_14_H_21_O]—(195.17 m/z). The loss of water for MCAs represents a common fragmentation pathway for these BAs, although neutral loss of hydrogen is also abserved in alpha MCA. Few product ions were detected in the spectra of BAs without a 12-hydroxyl group (Supplementary Material Fig. S1). The main fragments are the dehydrated forms for HCA [M–H–H_2_O] (389.3 m/z), CDCA (373.3 m/z), HDCA (373.3 m/z) and LCA (357.3 m/z). On the other hand, the low intensity of the dehydrated product of LCA (Supplementary Material Fig. S2) also confirmed that the 6-hydroxyl group and 7-hydroxyl group were more dehydrated than the 3-hydroxyl group.

The product ion, [M–H–H_2_O–CO]^−^ (345.3 m/z), was the major fragment for DCA together with the dehydrogenated form [M–H–H_2_O–CO–H_2_]^−^ (343.3 m/z) and the di-dehydrated form [M–H–H_2_O–CO–H_2_O]—(327.3 m/z). According with the described results, it has been recently highlighted^25^ that the dissociation mechanisms of BAs with a 12 hydroxy group involve the rotation of the carboxylate side chain and the subsequent rearrangements accompanied by proton transfer between 12-hydroxyl and 24-carboxyl groups.

**Figure 3:**
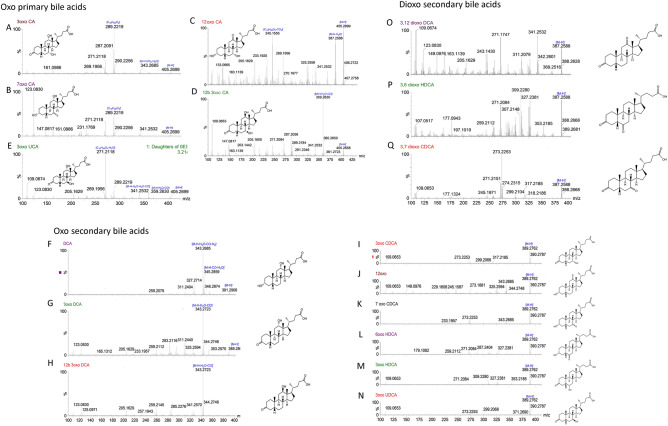
HR tandem MS spectra of the oxo metabolites of bile acids in the investigated vertebrates.

The systematic study of oxo-BA fragmentation was also addressed (Fig. 3). Oxo BAs showed an interesting fragmentation pattern, and these MS/MS spectra can be explained considering the results described above. The mono oxo derivatives from CA resulted in different fragmentation patterns, according to the position of the oxo group in the polycyclic core (Fig. 3A–D). In particular, the product ion [C_19_H_29_O_2_]^−^ (m/z 289.2) is one of the highest fragments for 3-oxo-CA, 7-oxo-CA and also for 3-oxo-UCA (Fig. 3A, B, E, respectively) as well as for CA, but it is almost completely absent for 12-oxo-CA (Fig. 3C), 12β-3oxo-CA (Fig. 3D) and 3-oxo-HCA. This suggests both the presence of a 12 hydroxy group^25^ and the 12 alpha orientation are necessary to obtain this fragmentation pattern. Indeed, 12β-3-oxo CA shows a different fragmentation pattern with the formation of the product ions [M–H–H_2_O–CO]^−^ (m/z 359.25) with its dehydrated form (m/z 341.24) (Fig. 3D). These discriminating ions are useful qualifiers for these specific mono oxo isomeric derivatives of CA.

Furthermore, the different relative abundances among ions could be used to enhance the selectivity of any analytical method for unconjugated BAs. Indeed, the product ion [C_19_H_29_O_2_]^−^ (m/z 289.2) is the most abundant for 3-oxo-CA (Fig. 3A) and 7-oxo-CA (Fig. 3B); while the dehydrated form [C_19_H_27_O]^−^—is the main product for 3-oxo-UCA (Fig. 3E).

Mass spectra at high collision energy (45 eV) of DCA and the oxo-DCAs (Fig. 3F–H) with a 12 alpha or beta hydroxy group confirmed the presence of the product ion [M–H–H_2_O–CO]^−^ (343.3 m/z) as the main fragment. In contrast, the DCA oxo metabolite at position 12 shows a low signal of this product ion (Fig. 3J).

The typical fragment [M–H–H_2_O]^−^ (m/z 371) and its dehydrated form (m/z 353) are only slightly detectable for secondary BAs without 12-hydroxyl groups, such as 3-oxo-CDCA, 7-oxo-CDCA, 6-oxo-HDCA, 3-oxo-HDCA, and 3-oxo-UDCA (Fig. 3I, K–N).

Regarding dioxo derivatives of the secondary BAs, few detectable product ions were determined. The ion [M–H_2_O–CO]^−^ (m/z 341) was detectable only for 3,12-dioxo-DCA (Fig. 3O), the product ion [C_23_H_35_O]^−^ (327 m/z) and the dehydrated form (309 m/z) were specific for 3,6-dioxo-HDCA (Fig. 3P), while the product ion [C_21_H_33_O_2_]^−^ (317 m/z) and the fragment [C_21_H_33_O_2_–CO_2_]^−^ (273 m/z) were specific for 3,7-dioxo-CDCA (Fig. 3Q).

The tandem mass spectra of LCA and its oxo derivative (Supplementary Material Fig. S2) show a slightly different fragmentation pattern. Dehydrated product ion [M–H_2_O]^−^ is not present for the 3-oxo-LCA, while it is detectable for LCA, although with a low ionic abundance. This behaviour could be correlated with the absence of a hydroxy group in the polycyclic core.

### Method validation

Calibration curves showed coefficients of determination (r^2^) ≥ 0.995 for all the BA molecules. LOD values ranged from 5 to 10 ng/mL, while LOQ values ranged from 15 to 30 ng/mL for all compounds. The results are shown in Supporting Material (Table S1).

Precision and accuracy (intra,_inter day), expressed as CV% and bias%, respectively, were < 10% for all compounds at three different concentration levels (Supporting Material, Table S2).

Selectivity was evaluated by comparing standard solutions and fortified samples. These comparisons showed that there were no significant interferences due to the matrix components, confirming the good selectivity of the method. Compounds 6-oxo-HDCA and 3-oxo-HDCA were the only unresolved compounds,therefore they were quantified as sum.

The matrix-matched calibration curve slopes did not differ significantly from those in neat solution (*p* = n.s.) in the same concentration range.

The best results among extraction protocols were obtained with both isopropanol and sodium hydroxide extraction (Rec > 90%) for oxo BAs without significant differences among the different animal fecal samples. On the other hand, the SPE step was a longer sample handling that introduced more variability related to different analyte recoveries. For this reason, isopropanol was used for sample extraction, with the advantage of a low impact of pre-analytical errors The results are reported in the supporting material (Table S3).

### Bile acids in animal feces

**Figure 4:**
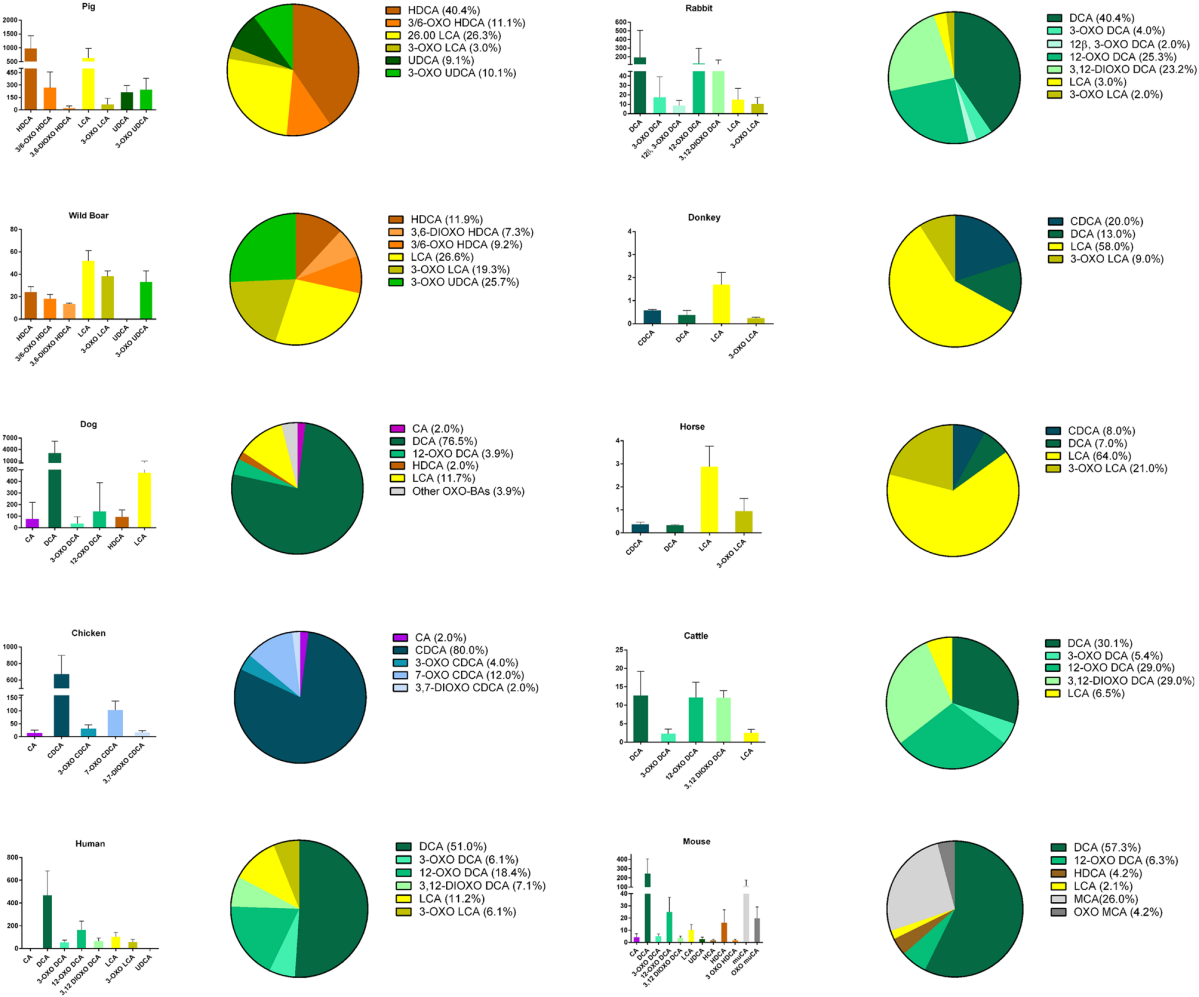
Column charts (absolute content in µg g^−1^) and pie charts (%) of the fecal bile acid and oxo bile acid compositions in the investigated vertebrates.

The method has been applied to evaluate the quali-quantitative composition of BAs in the feces of investigated animals and humans. The total content was reported as μg/g ± standard deviation (SD) of wet feces for each animal. The high variability of data is explained considering several factors that could affect the amount of BAs in feces: intestinal motility, daily variation, diet, age of the animal and genetic factors^26^. Indeed, the abundance percentage was also used to better compare BAs profiles (Fig. 4). Concentrations (μg/g _wet feces_), SD and relative abundances (%) of oxo-BAs and their precursors are reported in Tables S4A–J of the supporting material. Example of chromatogram for oxo BAs in fecal samples is reported in supporting material (Fig. S3).

Pigs (*sus scrofa domesticus*) are reported along with wild boar (*sus scrofa*) as belonging to the same family S*uidae* (Table S4A, B, Fig. 4). The detected BAs in feces are the dehydroxylated and oxo derivatives of HCA, the main BAs in pigs^27^. Indeed, HDCA covered a significant fraction of the total BA pool for pigs and wild boar, with relative abundances of 40% and 13%, respectively. On the other hand, the main difference between the two BA profiles is the percentage of oxo derivatives. The oxo-BAs cover more than 50% of the total in wild boar, while they cover approximately 25% in pigs. This difference is presumably due to a different intestinal metabolic process conditioned by the intestinal residence time, composition of the GM, diet, etc.

The dog (*Canis familiaris*) BAs profile presents a relative complexity, with several analytes detected (Table S4C, Fig. 4). According to the literature^28^, the main BAs are DCA and LCA (78% and 12%). Oxo metabolites were detected at very low percentages with respect to the other species, and as expected, the most abundant, although at low levels, was the 12-dehydrogenated derivative 12-OXO-DCA (4%).

The main BA in chicken (*Gallus gallus domesticus*) feces is CDCA (80%), a primary BA, in agreement with previous studies^29^ (Table S4D, Fig. 4). Oxidated derivatives of CDCA were detected, and one of them, 7-oxo-CDCA, was present at a relatively high concentration (12%).

Rabbits (Oryctolagus cuniculus) also present a complex BAs profile in terms of oxo-BAs (Table S4E, Fig. 4). This agrees with previously reported data for primary and secondary BAs^30^. According to our results, oxo derivatives of DCA have never been reported before, and the main detected BA is DCA (40%). The oxidated derivative of DCA represents the main fraction of the oxo-BAs, with a relative abundance of 23% for 3,12-dioxo-DCA. Oxo-derivatives of CDCA have been detected at concentrations below the LOQ.

The results for Donkey (*Equus africanus asinus*) are reported along with horses (*Equus ferus caballus*), since they belong belonging to the same family (Equidae). For both species, the absolute BA concentrations were very low (Table S4F, G, Fig. 4). The main BAs detected are LCA, CDCA and DCA. The profile is quite similar between these animals, but the oxo-derivatives of LCA are more abundant in donkeys than in horses, 21% and 9%, respectively.

Cattles are the most common type of large, domesticated ungulates. They are a member of the subfamily Bovinae and are commonly classified collectively as *Bos taurus*. According to literature,the main BAs detected in plasma and bile samples of cattle are CA and its glycine and taurine conjugates^31^. According to our results (Table S4H, Fig. 4), DCA and its oxo derivatives are the main BAs excreted in feces, covering more than 50% of the total content.

The mouse (Mus musculus) profile is quite different from the others, considering the presence of the named muricholic acids (MCAs).

The main quantified oxo BAs are the oxidated derivatives of DCA and MCA, covering more than 10% of the total pool (Table S4I, Fig. 4). All the oxo forms of MCA were not commercially available as reference standards; hence, quantification was carried out by monitoring the specific deprotonate molecular ion (m/z 405,2699) and using the standard solutions of 3-oxo-HCA as one of the available oxo-MCAs. This approximation could be considered reliable considering the comparable instrumental response that is normally recorded for BAs with similar position of the functional groups in the polycyclic core, especially for the deprotonated molecular ion [M–H]^+^.

To compare results between animals and humans, 10 samples from healthy human subjects, 5 males and 5 females, from 18 and 60 years old, were analysed. The results agreed with those reported in the literature and in our previous studies^9^. In particular, the main oxo derivatives are the oxo derivatives of DCA, covering approximately 35% of the total content of BAs in feces (Table S4J, Fig. 4).

The results described above are summarized in Table 2.

**Table 2:** Species-specific metabolic fingerprint from acidic sterolbiomes in animals and humans.

	Chickens	Donkeys	Horses	Rabbits	Pigs	Boars	Dogs	Cattle	Mice	Humans
CA	●			○			●	○		
CDCA	†	●●	●	○			○			
DCA		●	●	†	○		†	†	†	†
HDCA				○	†	●●	●		●	
LCA	○			●	●●	†	●●	●	●	
MCA									●●	
UDCA	○			○	●		●	●		
12-oxo CA				○			●			
3-oxo CDCA	●									
7-oxo CDCA	●●						○			
3,7-dioxo CDCA	●						○			
3-oxo DCA	○	†		●			○			●
(12β)3-oxo DCA				○						●
12-oxo DCA	○			○			●			●●
3,12-dioxo DCA				●	○		○			●
3-oxo HCA										
3/6-oxo HDCA				○	●●	●●				
3,6-dioxo HDCA				●	○	●				
3-oxo LCA	○	●	†	●	●	●●	○			
oxo MCA									●	
3-oxo UDCA			●	○	●	●●				○

○ corresponds to a relative abundance < 1%, ● corresponds to a relative abundance < 10%, ●● corresponds to a relative abundance higher than 10%, † is used to highlight the most abundant compound.

### Gut microbiome structure—fecal BA composition relationship

Fecal samples from 6 different farming animals were collected and analyzed (horse, donkey, pig, dog, chicken and rabbit). After the sequencing process of the V3-V4 hypervariable region of the 16S rRNA gene and bioinformatics pipeline, a total of 429 868 high-quality reads were obtained (mean per sample ± SD, 9 769 ± 1700). Sequences were clustered into 2 828 operational taxonomic units (OTUs) at 99% identity.

**Figure 5:**
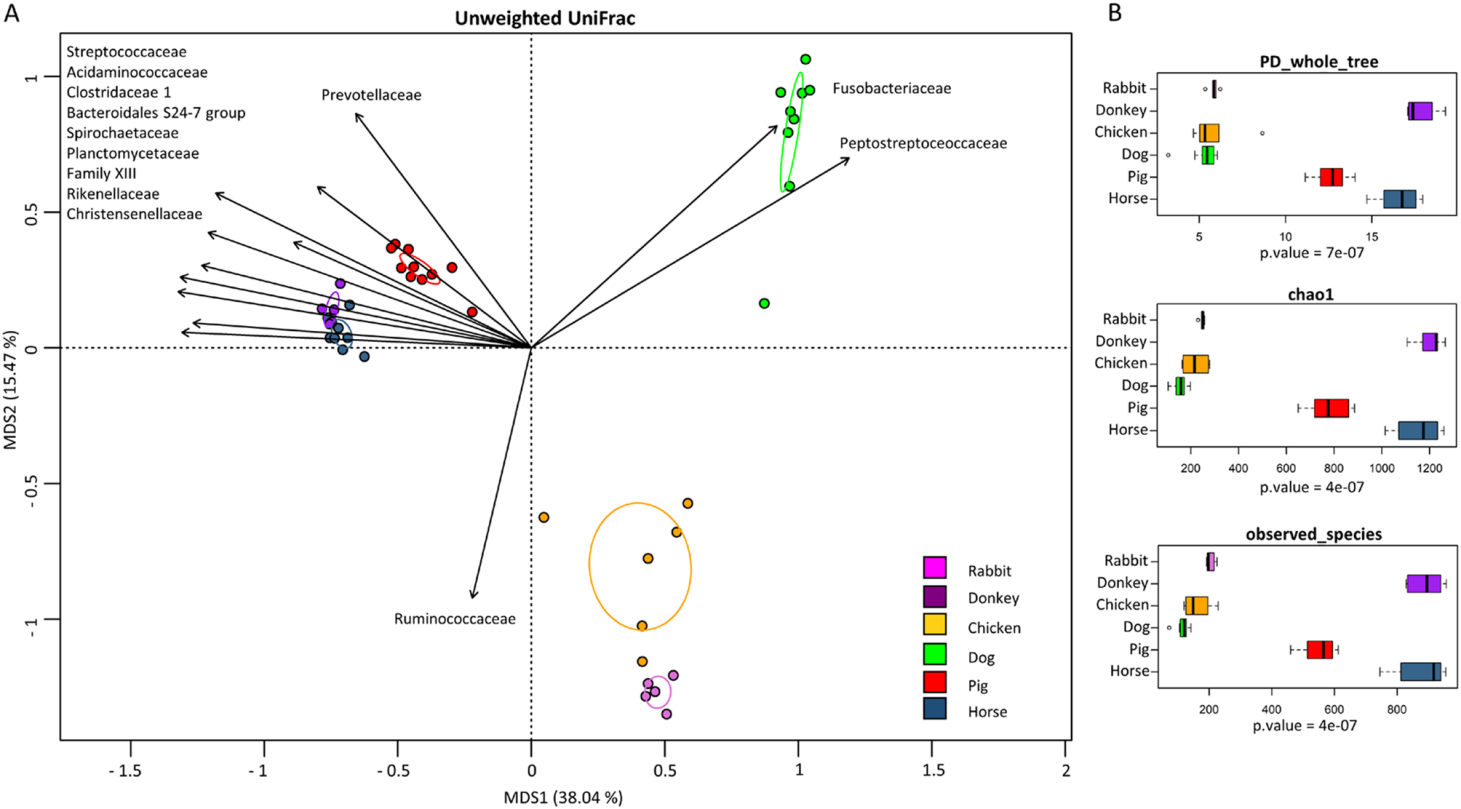
Diversity of the farming animals’ gut microbiota. PCoA of the unweighted UniFrac distances showing (**A**) all samples, a significant separation among animal groups was observed (permutation test with pseudo-F ratios (Adonis); *p* < 0.001). Black arrows were obtained by fitting the relative abundance of bacterial families for each sample within the ordination space (function envfit of the vegan R package). (**B**) Boxplots showing alpha diversity values according to the different metrics: Faith's phylogenetic diversity (PD_whole_tree), Chao1 index and OTU species count (observed_species). All metrics showed a significantly higher gut microbiota diversity for Donkey, Pig and Horse (Wilcoxon rank-sum test; *p* < 0.05).

To assess whether each animal had a specific gut microbiota profile, a principal coordinates analysis (PCoA) based on unweighted UniFrac distance was carried out. As expected, each animal clustered apart from the others (Fig. 5A), highlighting the specificity of the gut microbiota composition in the different farming animals (permutation test with pseudo-F ratios, P-value ≤ 0.001). Regarding intraspecies gut microbiome diversity (alpha diversity), all metrics showed significant differences among animal groups (Kruskal–Wallis test; *p* < 0.05). Specifically, horses, donkeys and pigs showed higher diversity values in the three metrics used (observed_species, PD_whole_tree and Chao1 index) compared to rabbits, chickens and dogs, which showed lower values, as assessed by the Wilcoxon rank-sum test across groups (*p* < 0.05) (Fig. 5B).

Specificities of the gut microbiota composition at the phylum, family and genus levels are shown in the Supplementary material (Fig. S4). At the phylum level, the most abundant taxon for all animals was Firmicutes, which represented approximately 69% of the whole intestinal bacterial ecosystem. At the family and genus levels, the composition of an animal’s bacterial ecosystem varies across different groups (Fig. S4), with host-specific patterns of dominant and subdominant families and genera.

**Figure 6:**
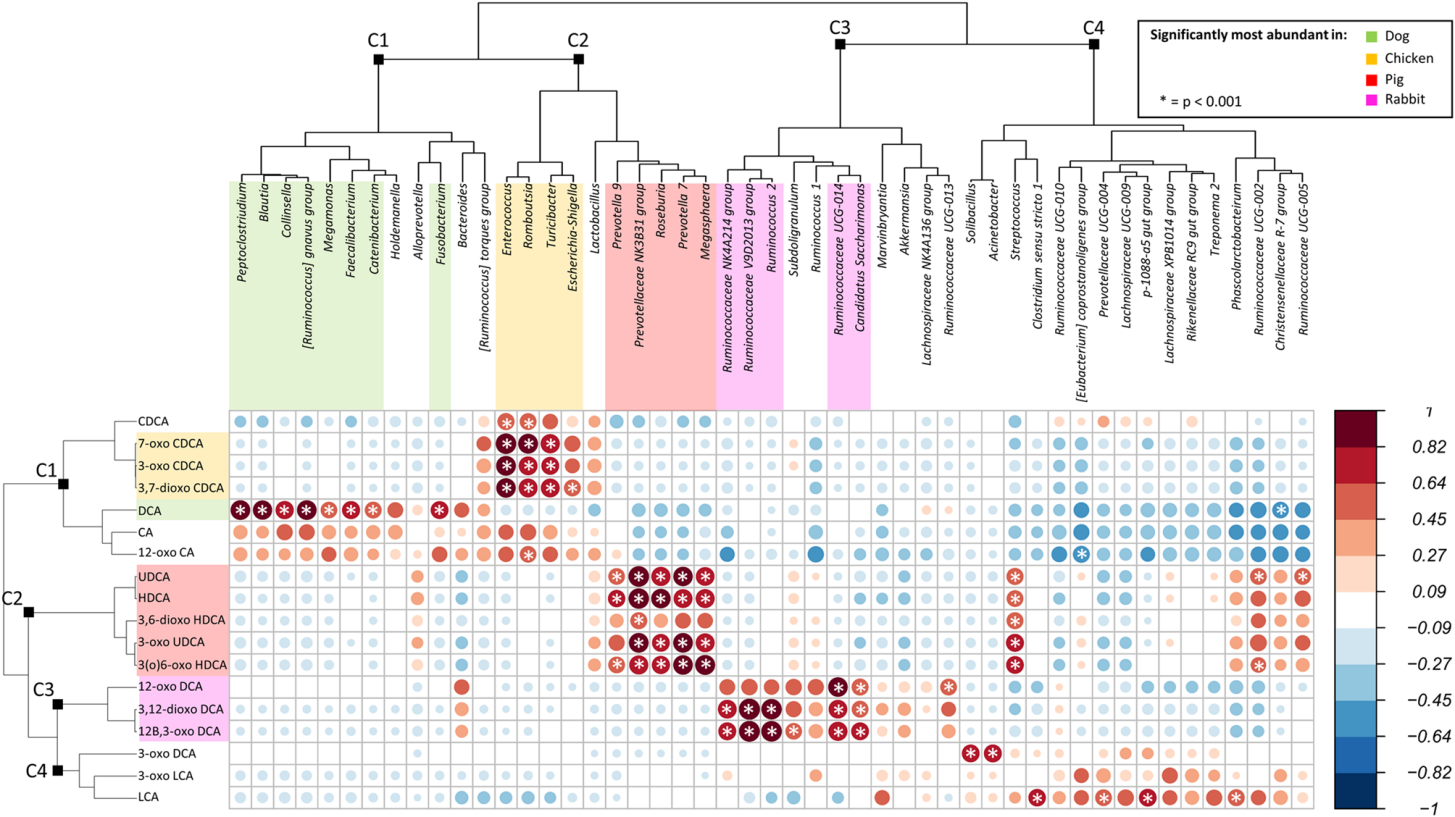
Correlation between animal gut microbiota components and BAs. The color is according to the Pearson correlation coefficients; positive correlations are shown in red, while negative correlations are depicted in blue. The color intensity and the size of the circle are proportional to the correlation values. In addition, a significant correlation was highlighted by a white asterisk in the centre of each circle (*p* ≤ 0.001). BA and bacterial genus correlation profiles were clustered using a hierarchical clustering approach, using the profile Pearson correlation value as a similarity measure and Ward’s linkage method. The obtained clusters were represented by dendrograms at the top and left margins of the correlogram. Within each cluster, both BAs and bacterial genera were coloured according to the colour legend in the top right of the figure to highlight the significantly most abundant genus or BA in a specific animal species (see Supplementary Material Figs. S4 and S5).

Next, we sought the Pearson correlation coefficients between microbiome genera and BAs in the comprehensive dataset from all farming animals. Only genera with a relative abundance ≥ 1.5% in at least two samples were considered. In Fig. 6, we provide the clustering of the abundance profiles obtained for the different taxa and BAs. Clustering analysis was performed by applying the hierarchical clustering method, using the Pearson correlation value between the taxa (or BAs) correlation profiles as distance. Significant correlations are shown as white asterisks (*p* ≤ 0.001).

**Figure 7:**
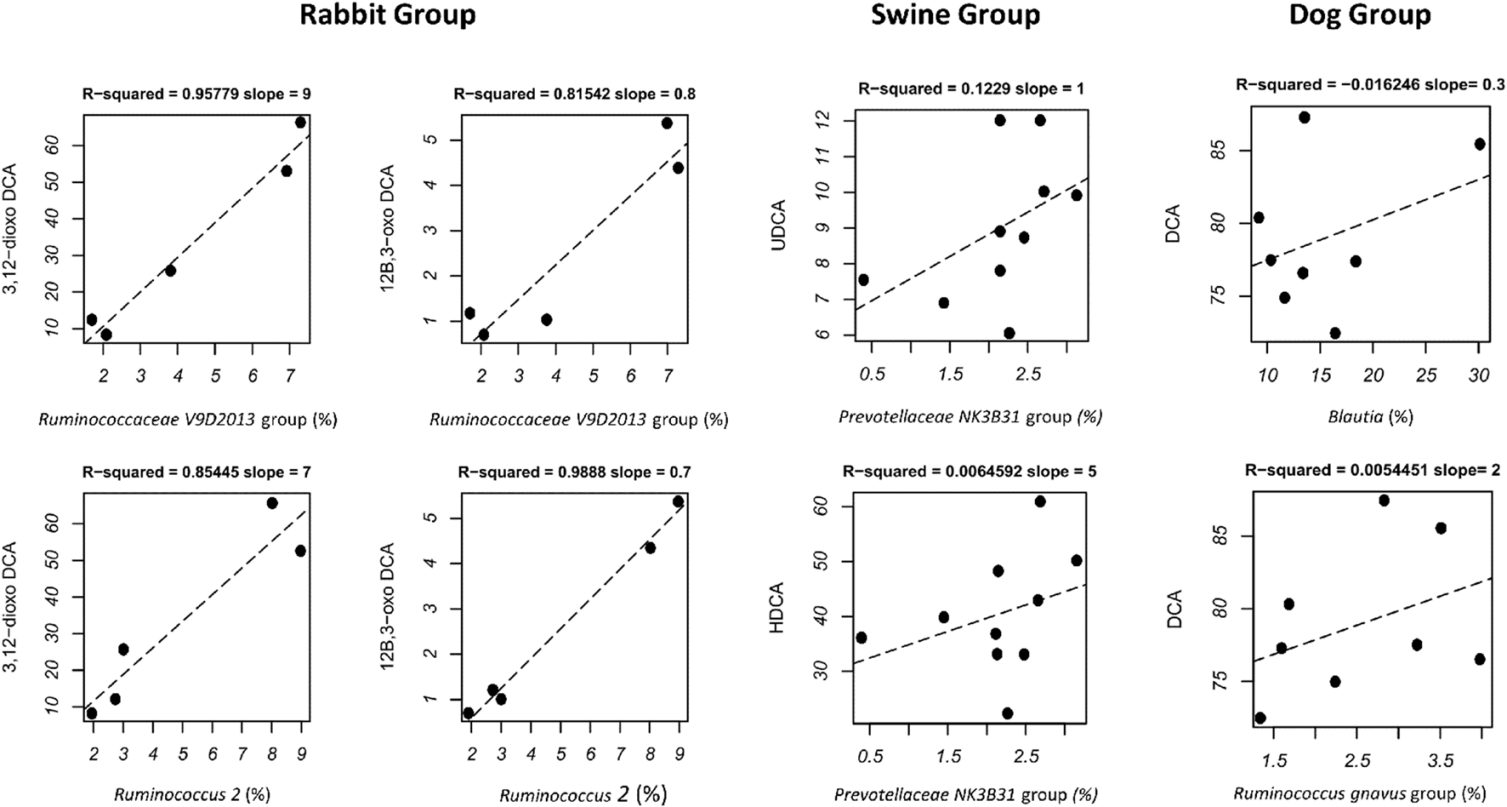
Linear regression plots showing positive associations between the relative abundances of gut genera and BAs in different farming animals.

This approach allowed the identification of 4 bacterial clusters showing a characteristic correlation profile with BAs. In particular, cluster 1, including microorganisms such as *Peptoclostridium*, *Blautia*, *Collinsella*, *[Ruminococcus] gnavus* group, *Megamonas*, *Fecalibacterium* and *Catenibacterium,* was positively correlated with DCA, CA and 12-oxo-CA. Members of cluster 2 foresee two groups of microorganisms, the first including *Enterococcus*, *Romboutsia*, *Turicibacter* and *Escherichia Shigella* and correlating positively with 7-oxo-CDCA, 3-oxo-CDCA and 3,7-dioxo-CDCA, and a second group of microorganisms (*Prevotellaceae* NK3B31 group, *Roseburia*, *Prevotella* 7 and *Megasphera*) correlating with a different set of BAs, such as UDCA, HDCA, 3,6-dioxo-HDCA, 3-oxo-UDCA and 3(o)6-oxo-HDCA. Finally, cluster 3, encompassing members of the *Ruminococcaceae* NK4A214 group, *Ruminococcaceae* V9D2013 group, *Ruminococcus* 2, *Ruminococcaceae* UCG-014 and *Candidatus Saccharimonas*, was positively correlated with 12-oxo DCA, 3,12-dioxo-DCA and 12β,3-oxo-DCA, while cluster 4 included a heterogeneous group of gut components showing a sparse pattern of positive and negative correlations with different BAs. Interestingly, microbial clusters 1–3 were dominated by genera that have been previously shown to characterize the gut microbiome from different farming animals (Figs. S5, S6) (Kruskal–Wallis test; *p* < 0.05). In particular, cluster 1 was dominated by microorganisms characterizing the dog’s gut microbiome, while the first and second groups of cluster 2 and cluster 3 included microorganisms characteristic of the chicken, pig and rabbit gut microbiomes, respectively. To strengthen these observations, for the different farming animals, we calculated one to one linear regression between the abundance of the specific gut microbiome components and the load of each BA in the different subjects. Figure 7 reports all the positive associations.

In particular, strong positive associations (r^2^ of 0.8) were obtained in rabbit for members of the family *Ruminococcaceae* (*Ruminococcaceae V9D2013 group, Ruminococcus 2*) and the BAs 3,12-dioxo-DCA and 12β,3-oxo DCA. Tendencies toward positive associations have been observed for swine, where the family *Prevotellaceae* NK3B31 associates with UDCA and HDCA, and for dogs, where both the *Ruminococcus gnavus* group and *Blautia* are associated with DCA abundance.

## Discussion

Quali-quantitative analysis of BAs, especially in biological matrices such as plasma and bile, has been extensively reported for many decades because of their biological multipotential role and for their implications in human physiological and pathological states. Several methods have been also reported for BA analysis in fecal samples^9,32^. Recently the determination of the fecal “acidic steroid profile” and the correlation with the GM^33^ has been gaining more attention by the specific community. In fact, the composition of the GM plays a pivotal role in the metabolism of BAs in the intestine, with the formation of numerous derivatives, including oxo-BAs.

In this scenario, we have developed a comprehensive LC–MS method for the analysis of 28 BAs, including 21 oxo-BAs^9^. Great effort has been made to establish an efficient extraction protocol for heterogeneous fecal samples, such as those from animals with different diets and digestive systems^34^. In fact, the water, fat and fiber contents can especially interfere with recovery rates. In this study, for the first time, a single extraction protocol (isopropanol solid–liquid extraction) was optimized, which allowed us to quantify BAs and oxo-BAs present in different concentration orders of magnitude and in different fecal matrices. Especially the variable water content among the samples did not affect the recovery reproducibility for oxo-BAs and BAs.

This method allowed us to determine the same BAs with the addition of MBAs in a short time with high selectivity due to the high-resolution MS. The analysis time was shortened up to 5 times compared to previous methods for oxo-BAs, maintaining, if not improving, the resolution of the chromatographic peaks and the selectivity by using high resolution.

Careful evaluation of the oxo-BA fragmentation patterns improved the specificity of these analytical methods, considering the similar chromatographic behaviour of the isobaric compounds analysed. Specific product ions and different ion abundances are useful tools to better discriminate among oxo bile acids with the same molecular weight and close retention time in columns. The key role of the 12-hydroxy group on the fragmentation patterns, supposed to be correlated to proton transfer between 12-hydroxyl and 24-carboxyl groups^25^, has been also confirmed in oxo-BA spectra.

To our knowledge, this is the first time that an almost complete profile of BAs and their metabolites produced by GM has been characterized in fecal samples by UPLC-HR-MS. In addition, the method was applied for the first time to the characterization of the "acidic steroid prolife" of 10 vertebrates, including humans. We focused our investigation on animals that have lived with humans for millennia for the purpose of breeding, work or companionship. Among the animals analyzed, we had 9 mammals, both herbivorous and omnivorous, and a bird.

Our study not only confirmed the peculiar profile of fecal BAs for each animal^3^ but also highlighted for the first time that the composition of the fecal oxo-BA GM metabolites differs for each species, even if belonging to the same family.

The profile of fecal BAs and oxo-BAs was then correlated to the composition of the intestinal microbiota to shed light on a possible microbiome-host co-metabolic network with species-specific declinations.

As expected, several genera positively correlated with BAs^35^, such as the Ruminococcus gnavus group, Ruminococcus 2, Collinsella, Fecalibacterium and Roseburia, including taxa possessing dehydroxylases and dehydrogenases able to metabolize various BAs. A complete list of bacteria possessing dehydroxylases and dehydrogenases able to metabolize various BAs is reported in the supplementary material (Table S5).

These correlations and profiles can only be the first step towards a deep correlation between microbiota and the “acidic steroid profile”, respecting the role of these hormone-like molecules within all living organisms.

Our findings demonstrate for the first time that the species-specific pattern of gut microbiome components explains a corresponding species-specific profile of BAs, highlighting the host-specific declinations of the BA microbiome-host co-metabolic asset. On the other hand, a variation in the BA profile in a given host can mirror a dysbiotic change in the core gut microbiome, suggesting the possibility of exploiting the profile of BAs as a proxy for a species-specific healthy gut microbiome.

Evidence of a correlation between the composition of GM and the "acidic sterolbiome" is important in the microbiota-driven production of metabolites (i.e., oxo-BAs), not only for excretion but also for their role as a potential ligand (agonist) to the FXR receptor, and in general for a better understanding of pathological events involving the liver and starting and connected with the intestine in the so-called gut-liver axis.

Minor BA fecal constituents at trace levels could be potentially present such as glycine/taurine-conjugated BAs, glucuronides, sulfate BAs or other metabolites under physiological and pathological conditions^36^. Future developments may include a broader class of compounds found especially in other mammals and vertebrates, for example, to investigate pathologic conditions affecting the intestinal tract of animals.

## Conclusion

The analytical method reported was developed and fully validated for the analysis of up to 30 compounds in feces, including 21 oxo-BAs. Careful evaluation was performed to determine the best extraction procedure for these compounds in fecal samples from different animals. The fecal oxo-BA profile was determined and reported for several farming animals for the first time in the literature. This analytical method is a powerful tool to better understand the key role of acid steroid compounds largely present in the intestinal tract of vertebrates. According to this purpose, the gut microbiota in farming animals was evaluated, allowing us to reconstruct species-specific associations between the BAs profile and specific GM components.

## Supplementary Information

Supplementary Information.
