# Supplementary material for: Analysis of fecal bile acids and metabolites by high resolution mass spectrometry in farm animals and correlation with microbiota
Source: Sci Rep. 2022 Feb 21;12:2866. doi: 10.1038/s41598-022-06692-9 (PMC8861013; doi:10.1038/s41598-022-06692-9)
Supplement: Supplementary file 1 — Supplementary Information. [file 41598_2022_6692_MOESM1_ESM.docx]

ANALYSIS OF FECAL BILE ACIDS AND METABOLITES BY HIGH RESOLUTION MASS SPECTROMETRY IN FARM ANIMALS AND CORRELATION WITH MICROBIOTA

Emanuele Porru^1^, Daniel Scicchitano^2^, Nicolò Interino^1^, Teresa Tavella^2^, Marco Candela^2^, Aldo Roda^1,3^, Jessica Fiori^1,4^

1 Department of Chemistry “G. Ciamcian”, University of Bologna, Bologna 40126, Italy

2 Department of Pharmacy and Biotechnology, University of Bologna, Via Belmeloro 6, 40126 Bologna, Italy

3 INBB-Biostructures and Biosystems National Institute, Viale delle Medaglie d'Oro 305 - 00136 Roma

4 Interdepartmental Center for Industrial Research–CIRI-MAM, University of Bologna, Bologna, Italy

**Supplementary material**

**Supplementary Figure S1.** HR tandem mass spectra of the primary bile acids CA, HCA, and muCAs.





**Supplementary Figure S2.** HR tandem mass spectra of lithocholic (LCA) acid its oxo derivative.

**
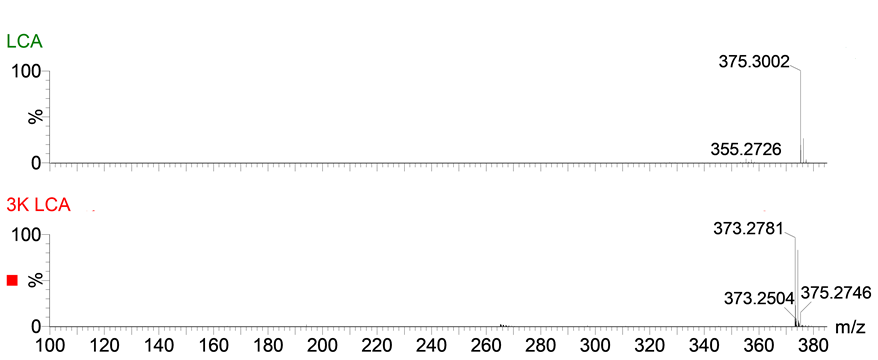
**

**Supplementary Figure** S**3** TIC of the oxo-BAs in chicken samples. 3α-hydroxy-7-oxo-cholan-24-oic acid (1), 7α-hydroxy-3-oxo-cholan-24-oic acid (2), 3,7-dioxo-cholan-24-oic acid (3), 3-oxo-cholan-24-oic acid (4).





**Supplementary Figure** S**4** Animal’s gut microbiota composition. Bar plot summarizing the microbiota composition at Phylum, Family and Genus level of animal’s faeces. Only phyla with a relative abundance ≥ 0.5% in at least 1 sample, families with relative abundance ≥ 1.5% in at least 1 sample and genera with a relative abundance ≥ 1.5% in at least 3 samples are represented.


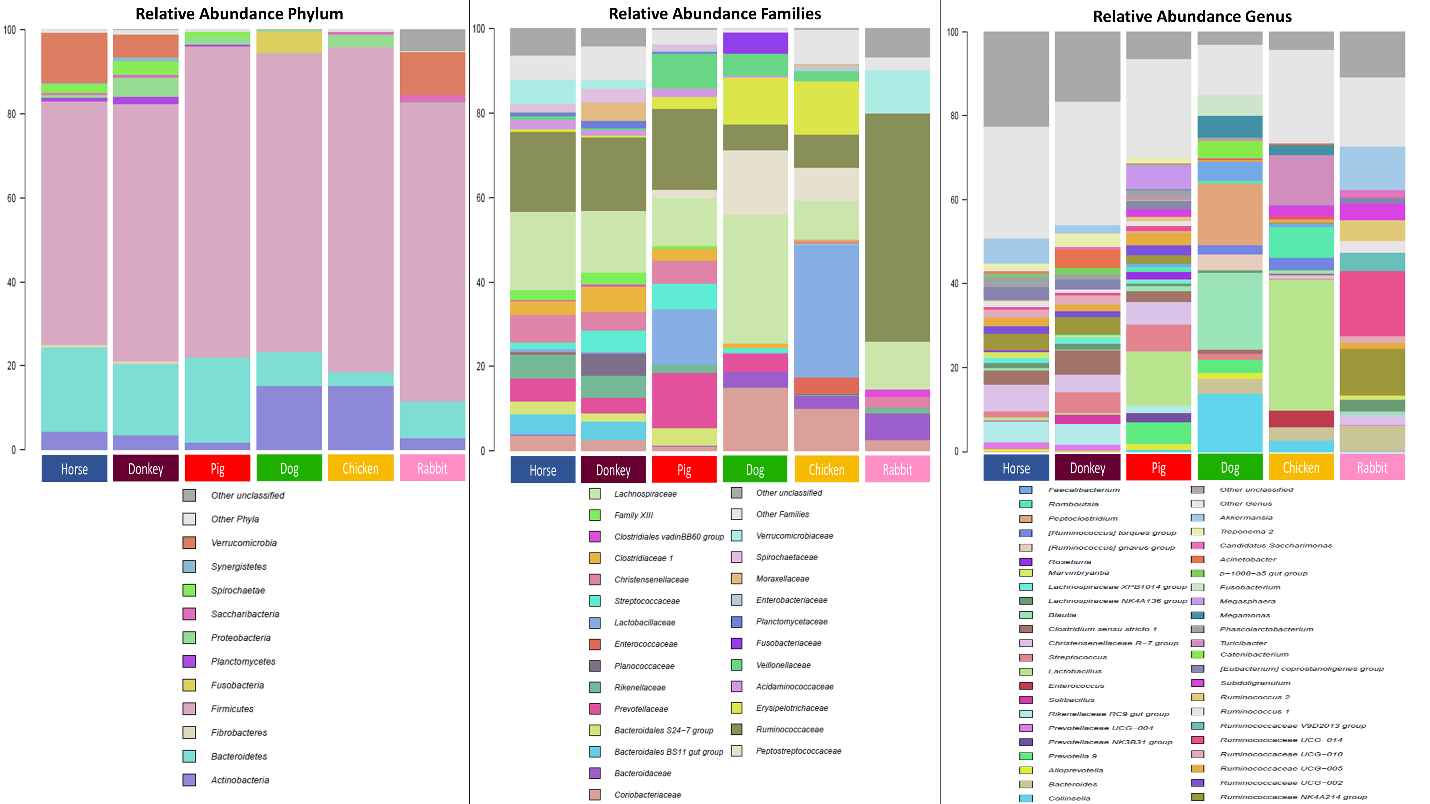


**Supplementary Figure S5**. Distributions of relative abundance of genera that showed a significant positive correlation with BAs. Kruskall-Wallis test with p ≤ 0.5, highlight that this genus is significant most abundant in a specific animal group. The central box of each dataset represents the distance between the 25th and the 75th percentiles. The median between them is marked with a black line.


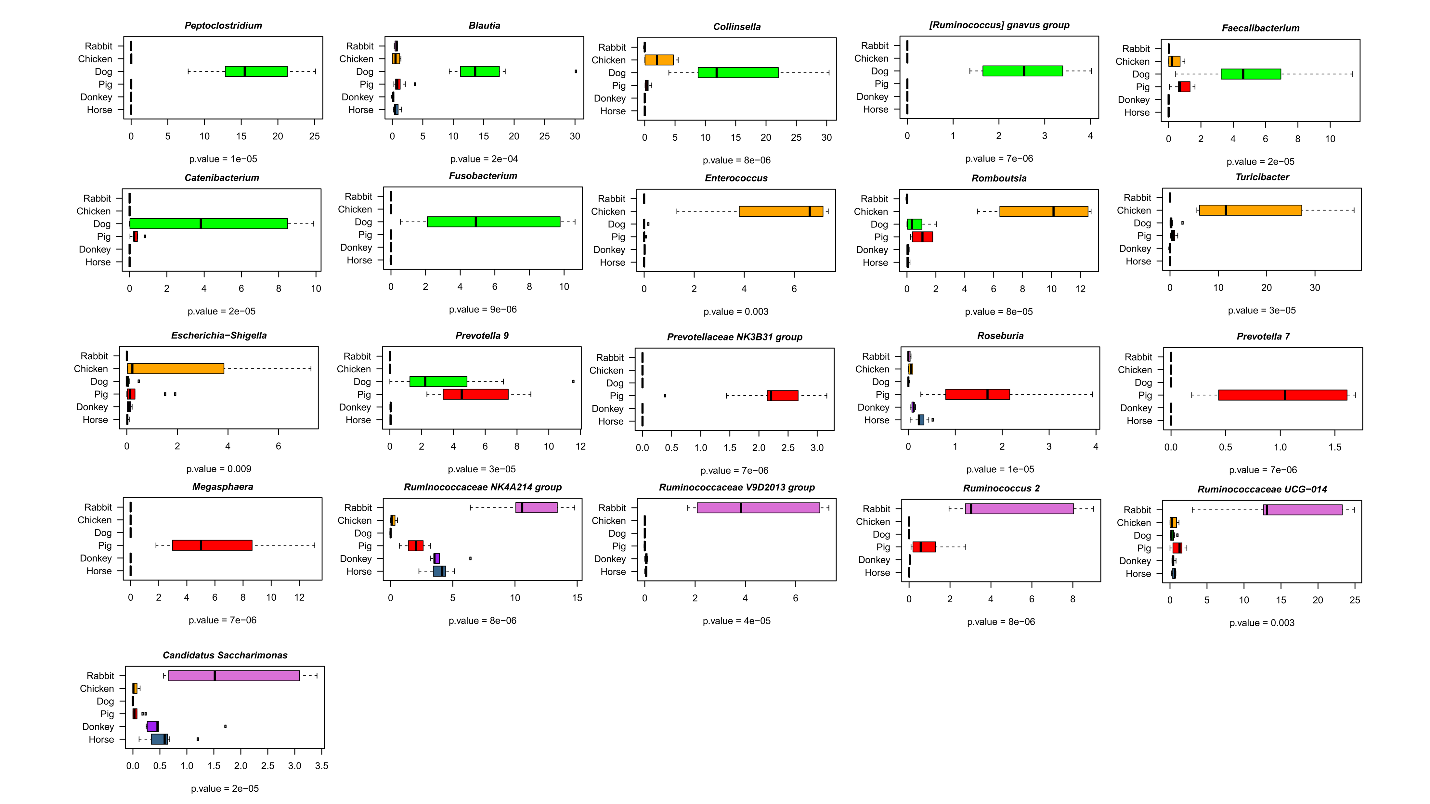


**Supplementary Figure S6**. Distributions of relative abundance of BAs that showed a significant positive correlation with bacterial genera. Kruskall-Wallis test with p ≤ 0.5, highlight that BA is significant most abundant in a specific animal group. The central box of each dataset represents the distance between the 25th and the 75th percentiles. The median between them is marked with a black line.


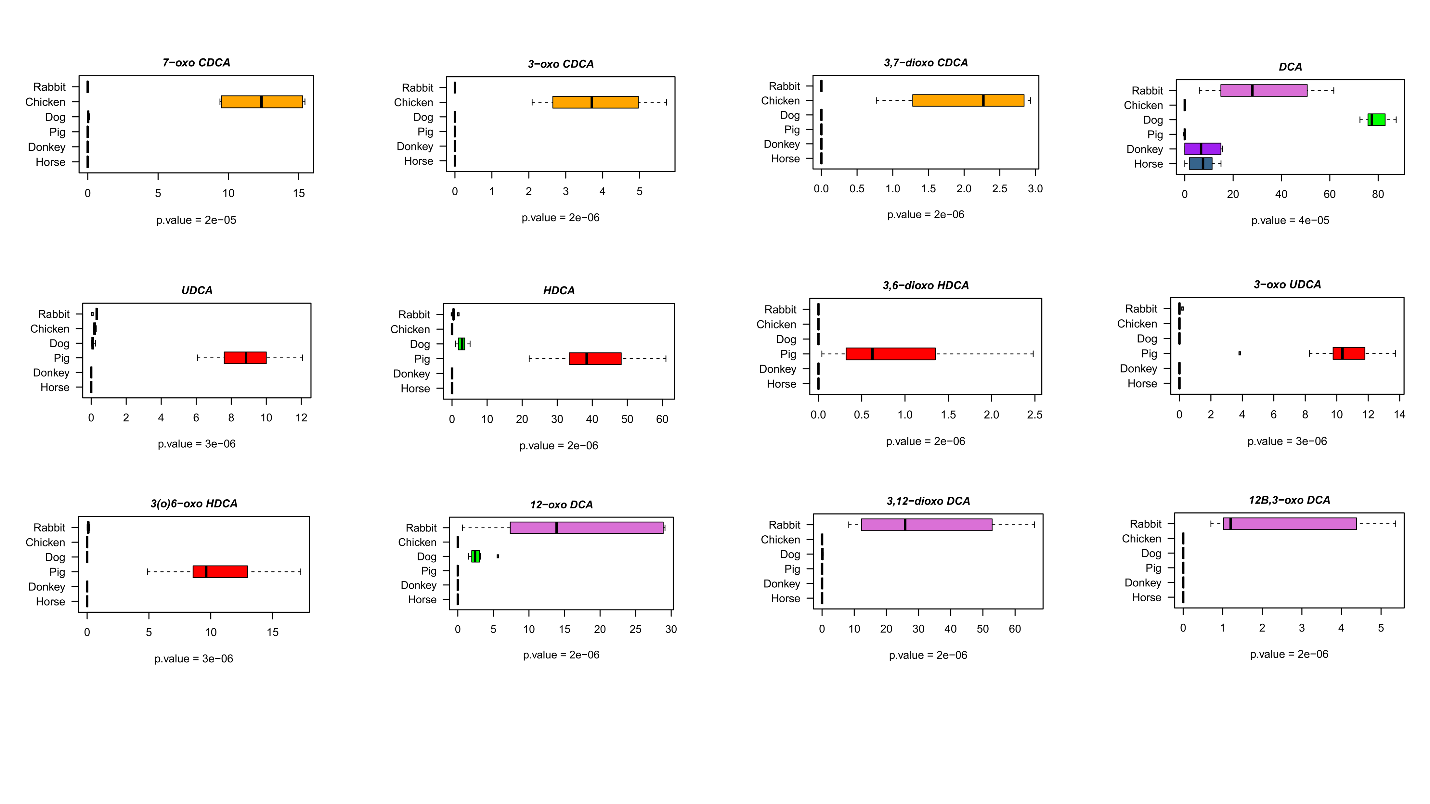


**Supplementary Table S1.** LOD and LOQ values of the developed method for all the investigated compounds.

|  | LOD (ng/mL) | LOQ (ng/mL) |
| --- | --- | --- |
|  |  |  |
| 7α,12α-dihydroxy-3-oxo-5β-cholan-24-oic acid | 10 | 30 |
| 7α,12β-dihydroxy-3-oxo-cholan-24-oic acid | 10 | 30 |
| 3α,12α-dihydroxy-7-oxo-cholan-24-oic acid | 10 | 30 |
| 3α,7α-dihydroxy-12-oxo-cholan-24-oic acid | 10 | 30 |
| 7α-hydroxy-3-oxo-cholan-24-oic acid | 5 | 15 |
| 3α-hydroxy-7-oxo-cholan-24-oic acid | 5 | 15 |
| 3,7-dioxo-cholan-24-oic acid | 5 | 15 |
| 12α-hydroxy-3-oxo-cholan-24-oic acid | 5 | 15 |
| 12β-hydroxy-3-oxo-cholan-24-oic acid | 5 | 15 |
| 3α-hydroxy-12-oxo-cholan-24-oic acid | 10 | 30 |
| 3,12-dioxo-cholan-24-oic acid | 10 | 30 |
| 3-oxo-cholan-24-oic acid | 1 | 10 |
| 7β-hydroxy-3-oxo-cholan-24-oic acid | 10 | 30 |
| 7β,12α-dihydroxy-3-oxo-cholan-24-oic acid | 5 | 15 |
| 3α,6α-dihydroxy-7-oxo-cholan-24-oic acid | 5 | 15 |
| 3α-hydroxy-6,7-dioxo-cholan-24-oic acid | 10 | 30 |
| 3,7,12-trioxo-cholan-24-oic acid | 10 | 30 |
| 3α-hydroxy-6-oxo-cholan-24-oic acid | 10 | 30 |
| 3,6-dioxo-cholan-24-oic acid | 10 | 30 |
| 6α-hydroxy-3-oxo-cholan-24-oic acid | 10 | 30 |
| 6α,7α-dihydroxy-3-oxo-cholan-24-oic acid | 5 | 15 |
| 3α,7α,12α-trihydroxy-5β-cholan-24-oic acid (CA) | 10 | 30 |
| 3α,6α,7α-trihydroxy-5β-cholan-24-oic acid (HCA) | 10 | 30 |
| 3α,7α-dihydroxy-5β-cholan-24-oic acid (CDCA) | 5 | 15 |
| 3α,12α-dihydroxy-5β-cholan-24-oic acid (DCA) | 5 | 15 |
| 3α,6α-dihydroxy-5β-cholan-24-oic acid (HDCA) | 10 | 30 |
| 3α,7β-dihydroxy-5β-cholan-24-oic acid (UDCA) | 10 | 30 |
| 3α-hydroxy-5β-cholan-24-oic acid (LCA) | 1 | 10 |

**Supplementary Table S2.** Precision (CV%) and accuracy (bias%) at three concentration level for each BA investigated.

|  | Intraday measurement (n = 3) | | | | | | Interday measurement (n = 9) | | | | | |  |
| --- | --- | --- | --- | --- | --- | --- | --- | --- | --- | --- | --- | --- | --- |
|  | QC_low_ | | QC_med_ | | QC_high_ | | QC_low_ | | QC_med_ | | QC_high_ | |  |
|  | Bias% | CV% | Bias% | CV% | Bias% | CV% | Bias% | CV% | Bias% | CV% | Bias% | CV% |  |
|  | |  |  |  |  |  |  |  |  |  |  |  |  |
| Trioxo CA | | 5 | 4 | 4 | 2 | 4 | 1 | 5 | 2 | 4 | 3 | 4 | 3 |
| 12β,3oxo-CA | | 5 | 3 | 3 | 2 | 5 | 2 | 4 | 3 | 4 | 3 | 2 | 3 |
| 3oxo-UCA | | 4 | 2 | 4 | 3 | 4 | 3 | 3 | 1 | 4 | 4 | 3 | 3 |
| 7oxo-HCA | | 5 | 2 | 4 | 2 | 3 | 3 | 2 | 1 | 5 | 2 | 4 | 1 |
| 7oxo-CA | | 3 | 3 | 2 | 2 | 1 | 3 | 2 | 2 | 3 | 2 | 4 | 1 |
| 12oxo-CA | | 4 | 2 | 4 | 3 | 4 | 2 | 4 | 2 | 5 | 3 | 5 | 2 |
| 3,7dioxo-CDCA | | 4 | 3 | 6 | 3 | 4 | 3 | 4 | 2 | 6 | 3 | 5 | 3 |
| 3oxo-HCA | | 6 | 4 | 4 | 3 | 4 | 2 | 5 | 2 | 4 | 2 | 4 | 2 |
| 3,12dioxo-DCA | | 5 | 3 | 5 | 3 | 5 | 2 | 4 | 3 | 4 | 3 | 4 | 2 |
| 3oxo-UDCA | | 4 | 3 | 5 | 3 | 5 | 4 | 3 | 2 | 4 | 3 | 4 | 3 |
| 3,6dioxo-HDCA | | 6 | 5 | 4 | 2 | 4 | 3 | 3 | 2 | 4 | 2 | 5 | 3 |
| 3oxo-HDCA | | 7 | 4 | 5 | 2 | 4 | 3 | 4 | 2 | 3 | 2 | 3 | 2 |
| 6 oxo HDCA | | 5 | 4 | 3 | 2 | 5 | 3 | 5 | 3 | 2 | 2 | 4 | 2 |
| 7-oxo CDCA | | 4 | 2 | 6 | 4 | 5 | 3 | 5 | 3 | 5 | 2 | 4 | 3 |
| 12β,3-oxo DCA | | 5 | 3 | 4 | 2 | 5 | 2 | 4 | 2 | 5 | 2 | 4 | 3 |
| 12-oxo DCA | | 6 | 4 | 4 | 2 | 4 | 2 | 4 | 3 | 3 | 3 | 5 | 3 |
| 6,7dioxo CA | | 4 | 3 | 2 | 2 | 3 | 3 | 3 | 2 | 5 | 2 | 4 | 3 |
| 3-oxo CDCA | | 5 | 2 | 5 | 4 | 4 | 2 | 4 | 3 | 4 | 3 | 5 | 3 |
| 3-oxo DCA | | 4 | 3 | 5 | 3 | 3 | 2 | 3 | 2 | 5 | 3 | 3 | 2 |
| 3-oxo LCA | | 5 | 3 | 4 | 3 | 4 | 3 | 5 | 3 | 3 | 2 | 4 | 3 |
|  | |  |  |  |  |  |  |  |  |  |  |  |  |
| A muCa | | 3 | 3 | 4 | 3 | 4 | 3 | 3 | 4 | 4 | 4 | 4 | 2 |
| B muCa | | 3 | 3 | 3 | 4 | 3 | 3 | 3 | 2 | 3 | 3 | 4 | 2 |
| HCA | | 4 | 2 | 4 | 3 | 5 | 3 | 5 | 2 | 4 | 2 | 6 | 3 |
| UDCA | | 4 | 3 | 4 | 3 | 6 | 4 | 5 | 2 | 3 | 2 | 3 | 2 |
| HDCA | | 6 | 2 | 4 | 3 | 5 | 3 | 4 | 2 | 3 | 2 | 5 | 3 |
| CA | | 4 | 2 | 4 | 3 | 5 | 3 | 5 | 2 | 4 | 2 | 6 | 3 |
| CDCA | | 4 | 2 | 4 | 2 | 4 | 3 | 5 | 3 | 5 | 3 | 4 | 2 |
| DCA | | 5 | 3 | 4 | 3 | 4 | 2 | 4 | 2 | 5 | 2 | 3 | 2 |
| LCA | | 5 | 2 | 5 | 3 | 3 | 2 | 5 | 3 | 4 | 3 | 6 | 4 |

**Supplementary Table S3.** Recovery (Rec%) for all the investigated compounds in all the animal faecal extracts at 2 ug/ml using isopropanol.

|  | Recovery (Rec % ± SD) n=3 | | | | | | | | | |
| --- | --- | --- | --- | --- | --- | --- | --- | --- | --- | --- |
|  | Human | Chicken | Pig | Wild Boar | Horse | Donkey | Mouse | Dog | Cattle | Rabbit |
| 7α,12α-dihydroxy-3-oxo-cholan-24-oic acid | 95,2  ± 0,8 | 96,3  ± 1,1 | 97,3  ± 1,4 | 96,9  ± 1,3 | 95,5  ± 1,2 | 99,3  ± 0,8 | 99,0  ± 2,0 | 97,3  ± 1,2 | 97,9  ± 0,9 | 97,4  ± 1,7 |
| 7α,12β-dihydroxy-3-oxo-cholan-24-oic acid | 97,5  ± 0,5 | 94,5  ± 1,3 | 98,5  ± 1,6 | 98,4  ± 2,3 | 97,9  ± 3,1 | 99,3  ± 1,1 | 98,9  ± 0,3 | 98,9  ± 2,1 | 99,0  ± 0,4 | 98,4  ± 0,8 |
| 3α,12α-dihydroxy-7-oxo-cholan-24-oic acid | 99,2  ± 0,9 | 97,5  ± 3,5 | 96,3  ± 2,4 | 98,6  ± 1,2 | 98,6  ± 1,4 | 97,5  ± 0,5 | 97,6  ± 1,2 | 98,6  ± 0,4 | 98,4  ± 1,9 | 99,3  ± 1,2 |
| 3α,7α-dihydroxy-12-oxo-cholan-24-oic acid | 98,7  ± 0,8 | 96,4  ± 1,1 | 96,4  ± 2,6 | 94,8  ± 1,2 | 99,6  ± 1,6 | 97,9  ± 2,3 | 98,5  ± 1,2 | 97,5  ± 1,5 | 99,1  ± 2,3 | 97,9  ± 1,2 |
| 7α-hydroxy-3-oxo-  cholan-24-oic acid | 95,5  ± 1,3 | 97,5  ± 231 | 98,9  ± 0,9 | 98,9  ± 1,8 | 97,4  ± 2,0 | 98,4  ± 2,1 | 97,2  ± 0,6 | 96,6  ± 0,6 | 99,7  ± 2,1 | 96,0  ± 0,9 |
| 3α-hydroxy-7-oxo-  cholan-24-oic acid | 98,4  ± 1,0 | 95,6  ± 1.6 | 97,3  ± 1,5 | 99,2  ± 0,9 | 97,6  ± 2,3 | 97,9  ± 2,1 | 99,1  ± 0,9 | 97,8  ± 2,4 | 94,2  ± 0,8 | 99,0  ± 2,2 |
| 3,7-dioxo-  cholan-24-oic acid | 98,5  ± 0,4 | 97,7  ± 1,6 | 99,3  ± 0,7 | 94,0  ± 1,3 | 94,7  ± 1,9 | 96,2  ± 2,1 | 98,9  ± 1,0 | 99,9  ± 2,0 | 96,7  ± 1,7 | 98,8  ± 1,7 |
| 12α-hydroxy-3-oxo-  cholan-24-oic acid | 97,5  ± 1,5 | 98,9  ± 1,2 | 95,9  ± 2,3 | 99,4  ± 0,9 | 99,6  ± 2,0 | 98,7  ± 2,9 | 96,6  ± 1,4 | 95,0  ± 0,9 | 98,7  ± 0,8 | 99,2  ± 1,7 |
| 12β-hydroxy-3-oxo-  cholan-24-oic acid | 98,8  ± 1,6 | 95,8  ± 0,9 | 95,0  ± 1,4 | 95,4  ± 1,3 | 93,6  ± 1,4 | 98,7  ± 1,9 | 99,1  ± 0,8 | 98,3  ± 2,2 | 97,2  ± 0,7 | 96,8  ± 1,1 |
| 3α-hydroxy-12-oxo-  cholan-24-oic acid | 95,4  ± 1,2 | 99,8  ± 3,3 | 95,0  ± 1,0 | 98,1  ± 2,0 | 97,4  ± 3,4 | 98,4  ± 3,1 | 96,7  ± 1,8 | 95,4  ± 1,2 | 99,4  ± 3,3 | 98,7  ± 3,4 |
| 3,12-dioxo-  cholan-24-oic acid | 95,7  ± 0,9 | 97,3  ± 0,4 | 99,3  ± 3,2 | 97,8  ± 1,3 | 95,3  ± 2,3 | 98,3  ± 1,9 | 99,7  ± 0,9 | 99,0  ± 1,5 | 97,8  ± 1,8 | 99,5  ± 2,3 |
| 3-oxo-  cholan-24-oic acid | 99,2  ± 3,5 | 97,9  ± 2,4 | 96,3  ± 1,8 | 98,4  ± 1,4 | 97,3  ± 0,9 | 99,9  ± 1,8 | 97,3  ± 1,3 | 97,1  ± 1,1 | 98,3  ± 0,4 | 99,1  ± 1,6 |
| 7β-hydroxy-  3-oxo-cholan-24-oic acid | 98,0  ± 2,2 | 96,5  ± 1,3 | 96,5  ± 2,6 | 96,2  ± 5,4 | 94,6  ± 2,6 | 96,8  ± 1,0 | 98,1  ± 1,6 | 97,1  ± 1,7 | 97,0  ± 4,2 | 97,6  ± 1,1 |
| 7β,12α-dihydroxy-  3-oxo-cholan-24-oic acid | 98,7  ± 0,6 | 96,9  ± 0,8 | 94,4  ± 1,3 | 98,9  ± 1,4 | 95,8  ± 1,2 | 97,4  ± 1,1 | 99,0  ± 0,3 | 93,7  ± 1,1 | 99,2  ± 2,1 | 99,4  ± 0,5 |
| 3α,6α-dihydroxy-7-oxo-cholan-24-oic acid | 95,7  ± 0,2 | 98,6  ± 2,8 | 96,2  ± 1,6 | 94,7  ± 2,1 | 96,7  ± 0,9 | 95,7  ± 1,3 | 97,5  ± 1,3 | 99,5  ± 0,8 | 96,2  ± 2,4 | 98,5  ± 1,3 |
| 3α-hydroxy-6,7-dioxo-cholan-24-oic acid | 97,2  ± 1,3 | 97,6  ± 1,0 | 98,6  ± 0,6 | 99,5  ± 2,4 | 95,8  ± 1,9 | 96,8  ± 2,9 | 99,7  ± 0,5 | 95,1  ± 0,5 | 94,3  ± 1,7 | 97,5  ± 0,8 |
| 3,7,12-trioxo-  cholan-24-oic acid | 97,0  ± 1,2 | 99,2  ± 0,8 | 94,7  ± 0,9 | 99,2  ± 2,4 | 94,8  ± 1,7 | 99,1  ± 1,4 | 96,5  ± 0,7 | 97,7  ± 0,9 | 98,9  ± 3,5 | 95,5  ± 2,1 |
| 3α-hydroxy-6-oxo-  cholan-24-oic acid | 95,4  ± 1,6 | 98,6  ± 1,3 | 96,7  ± 2,6 | 97,4  ± 1,7 | 95,7  ± 0,9 | 98,4  ± 1,2 | 97,2  ± 0,9 | 98,3  ± 1,1 | 97,9  ± 3,2 | 96,3  ± 2,6 |
| 3,6-dioxo-cholan-24-oic acid | 95,3  ± 3,5 | 99,2  ± 0,6 | 96,7  ± 2,4 | 99,9  ± 0,5 | 98,7  ± 0,7 | 97,8  ± 2,0 | 96,5  ± 2,0 | 97,1  ± 0,6 | 97,5  ± 0,8 | 98,4  ± 0,9 |
| 6α-hydroxy-3-oxo-  cholan-24-oic acid | 96,3  ± 1,1 | 97,3  ± 0,9 | 99,5  ± 2,4 | 98,4  ± 0,5 | 97,9  ± 0,8 | 99,5  ± 2,1 | 95,2  ± 0,7 | 99,4  ± 1,8 | 94,2  ± 3,4 | 99,0  ± 2,3 |
| 6α,7α-dihydroxy-3-oxo-cholan-24-oic acid | 98,6  ± 2,3 | 95,9  ± 1,3 | 97,9  ± 2,1 | 95,0  ± 3,1 | 97,4  ± 2,0 | 98,7  ± 1,7 | 99,7  ± 2,1 | 95,7  ± 0,9 | 96,8  ± 2,8 | 99,5  ± 2,4 |
| 3α,7α,12α-trihydroxy-5β-cholan-24-oic acid (CA) | 98,7  ± 0,5 | 98,9  ± 1,7 | 94,4  ± 2,3 | 96,7  ± 0,7 | 94,4  ± 1,9 | 96,4  ± 04 | 97,4  ± 2,1 | 96,9  ± 0,7 | 99,7  ± 1,9 | 98,2  ± 2,1 |
| 3α,6α,7α-trihydroxy-5β-cholan-24-oic acid (HCA) | 95,9  ± 1,6 | 98,2  ± 0,5 | 93,4  ± 0,7 | 98,6  ± 1,3 | 95,7  ± 2,7 | 96,7  ± 1,2 | 98,4  ± 2,1 | 95,7  ± 1,1 | 98,2  ± 2,4 | 97,5  ± 1,4 |
| 3α,7α-dihydroxy-5β-cholan-24-oic acid (CDCA) | 98,3  ± 0,4 | 99,1  ± 2,0 | 97,4  ± 0,7 | 96,8  ± 1,7 | 99,0  ± 1,4 | 95,1  ± 1,9 | 97,4  ± 3,1 | 93,5  ± 0,6 | 97,5  ± 1,0 | 99,2  ± 3,5 |
| 3α,12α-dihydroxy-5β-cholan-24-oic acid (DCA) | 95,8  ± 1,0 | 97,7  ± 2,2 | 97,9  ± 2,1 | 98,7  ± 1,4 | 94,5  ± 0,7 | 97,9  ± 2,1 | 95,8  ± 1,0 | 98,4  ± 1,7 | 99,2  ± 0,9 | 93,2  ± 1,4 |
| 3α,6α-dihydroxy-5β-cholan-24-oic acid (HDCA) | 99,5  ± 1,4 | 95,7  ± 1,4 | 97,8  ± 0,8 | 98,9  ± 3,1 | 95,9  ± 1,6 | 98,4  ± 2,0 | 97,0  ± 1,8 | 98,5  ± 0,5 | 99,5  ± 0,7 | 97,4  ± 2,2 |
| 3α,7β-dihydroxy-5β-cholan-24-oic acid (UDCA) | 98,2  ± 0,3 | 98,8  ± 3,3 | 100,5  ± 0,7 | 97,1  ± 2,4 | 98,2  ± 1,7 | 95,5  ± 0,8 | 96,4  ± 2,1 | 97,6  ± 3,1 | 94,1  ± 1,5 | 96,2  ± 3,0 |
| 3α-hydroxy-5β-  cholan-24-oic acid (LCA) | 95,0  ± 1,9 | 97,5  ± 2,0 | 99,0  ± 0,8 | 98,7  ± 1,3 | 97,4  ± 1,0 | 98,5  ± 0,8 | 99,0  ± 0,9 | 95,7  ± 0,9 | 95,9  ± 1,2 | 98,0  ± 0,5 |

**Supplementary Table S4A.** Bile acid concentration in Pig (*sus scrofa domesticus)*

| Bile acid | Concentration${}^{\dagger}$ | | SD${}^{\dagger}$ | Relative abundance (%) |
| --- | --- | --- | --- | --- |
| 3,6-DIOXO HDCA | 21 | 28 | | 1 |
| 3/6-OXO HDCA | 266 | 192 | | 11 |
| 3-OXO UDCA | 244 | 137 | | 10 |
| 3-OXO LCA | 65 | 76 | | 3 |
|  |  |  | |  |
| DCA | 0.49 | 0.54 | | <1 |
| HDCA | 971 | 465 | | 40 |
| LCA | 631 | 348 | | 26 |
| UDCA | 211 | 80 | | 9 |
| CDCA | 1.19 | 1.71 | | <1 |

**Supplementary Table S4B.** Bile acid concentration in Wild boar *(sus scrofa)*

| Bile acid | Concentration${}^{\dagger}$ | SD${}^{\dagger}$ | Relative abundance (%) |
| --- | --- | --- | --- |
| 3,6-DIOXO HDCA | 13.5 | 0.8 | 8 |
| 3/6-OXO HDCA | 18 | 4 | 10 |
| 3-OXO UDCA | 33 | 10 | 28 |
| 3-OXO LCA | 38 | 5 | 21 |
|  |  |  |  |
| HDCA | 24 | 5 | 13 |
| LCA | 52 | 9 | 29 |

**Supplementary Table S4C.** Bile acid concentration in dogs (*Canis familiaris)*

| Bile acid | Concentration ${}^{\dagger}$ | SD${}^{\dagger}$ | Relative abundance (%) |
| --- | --- | --- | --- |
| 7-OXO CDCA | 1.49 | 1.53 | <1 |
| 3-OXO LCA | 6,79 | 10.84 | <1 |
| 12-OXO CA | 3.96 | 8.48 | <1 |
| 12-OXO DCA | 141 | 247 | 4 |
| 3,12-DIOXO DCA | 6.86 | 13.99 | <1 |
| 3-OXO DCA | 35.33 | 59.71 | 1 |
|  |  |  |  |
| CA | 78.38 | 141.02 | 2 |
| CDCA | 9.46 | 10.46 | <1 |
| DCA | 3033 | 3150 | 78 |
| HDCA | 93.84 | 58.85 | 2 |
| LCA | 472 | 519 | 12 |
| UDCA | 9.13 | 19.16 | <1 |

**Supplementary** **Table S4D.** Bile acid concentration in Chicken (*Gallus gallus domesticus*)

| Bile acid | Concentration${}^{\dagger}$ | SD${}^{\dagger}$ | Relative abundance (%) | |
| --- | --- | --- | --- | --- |
| 3,7-DIOXO CDCA | 16.3 | 7.77 | 2 |  |
| 7-OXO CDCA | 103 | 34 | 12 |  |
| 3-OXO CDCA | 31.28 | 14.58 | 4 |  |
| 3-OXO LCA | 0.98 | 0.05 | <1 |  |
|  |  |  |  |  |
| CA | 14.2 | 11.9 | 2 |  |
| CDCA | 673.13 | 225.12 | 80 |  |
| LCA | 1.90 | 0.03 | <1 |  |
| UDCA | 1.78 | 0.97 | <1 |  |

**Supplementary Table S4E.** Bile acid concentration in Rabbit (Oryctolagus cuniculus domestic)

| OXO-BA | Concentration ${}^{\dagger}$ | SD${}^{\dagger}$ | Relative abundance (%) |
| --- | --- | --- | --- |
| 3/6 -OXO HDCA | 0.27 | 0.19 | <1 |
| 3-OXO LCA | 10.3 | 6.9 | 2 |
| 12BETA, 3-OXO DCA | 8.66 | 5.50 | 2 |
| 12-OXO DCA | 122 | 172 | 25 |
| 3,12-DIOXO DCA | 110 | 51.3 | 23 |
| 3-OXO DCA | 17.2 | 22.1 | 4 |
|  |  |  |  |
| CA | 1.31 | 1.58 | <1 |
| DCA | 192.11 | 314 | 40 |
| HDCA | 1.79 | 2.18 | <1 |
| LCA | 14.96 | 11.78 | 3 |
| UDCA | 0.86 | 0.46 | <1 |

**Supplementary Table S4F.** Bile acid concentration in Donkey (*Equus africanus asinus*)

| Bile acid | Concentration ${}^{\dagger}$ | SD${}^{\dagger}$ | | Relative abundance (%) |
| --- | --- | --- | --- | --- |
| 3-OXO LCA | 0.25 | 0.03 | 9 | |
|  |  |  |  | |
| CDCA | 0.58 | 0.04 | 20 | |
| LCA | 1.70 | 0.52 | 58 | |
| DCA | 0.38 | 0.20 | 13 | |

**Table S4G.** Bile acid concentration in Horse (*Equus ferus caballus*)

| Bile acid | Concentration | SD${}^{\dagger}$ | Relative abundance (%) |  |
| --- | --- | --- | --- | --- |
| 3-OXO LCA | 0.95 | 0.55 | 21 |  |
|  |  |  |  | |
| DCA | 0.33 | 0.02 | 7 |  |
| CDCA | 0.37 | 0.10 | 8 |  |
| LCA | 2.88 | 0.89 | 64 |  |

**Supplementary Table S4H.** Bile acid concentration in Cattle (Bos taurus)

| Bile acid | Concentration | SD${}^{\dagger}$ | Relative abundance (%) |
| --- | --- | --- | --- |
| 12-OXO DCA | 12.08 | 4.15 | 27 |
| 3,12 DIOXO DCA | 12.00 | 1.94 | 27 |
| 3-OXO DCA | 2.33 | 1.16 | 5 |
| 12-OXO CA | 0.53 | 0.30 | 1 |
|  |  |  |  |
| DCA | 12.68 | 6.52 | 28 |
| LCA | 2.48 | 0.99 | 6 |
| UDCA | 2.40 | 0.35 | 5 |
| CA | 0.15 | 0.15 | <1 |

**Supplementary Table S4I.** Bile acid concentration in Mice

| Bile acid | Concentration ${}^{\dagger}$ | SD${}^{\dagger}$ | Relative abundance (%) |
| --- | --- | --- | --- |
| 3,12-DIOXO DCA | 3,85 | 1,4 | 0,8 |
| 12-OXO DCA | 25,1 | 12,1 | 5,5 |
| 3-OXO DCA | 5,2 | 1,9 | 1,1 |
| 3-OXO LCA | 0,32 | 0,18 | 0,1 |
| OXO muCA* | 19,7 | 9,40 | 4,3 |
| 3-OXO HCA | 0,7 | 0,3 | 0,2 |
| OXO HDCA | 1,8 | 0,9 | 0,4 |
|  |  |  |  |
| CA | 4.31 | 2,78 | 0,9 |
| CDCA | 0,95 | 1,0 | 0,2 |
| DCA | 247,5 | 157,12 | 54,6 |
| HCA | 1,7 | 0,8 | 0,4 |
| HDCA | 16,13 | 10,71 | 3,6 |
| LCA | 10,11 | 4,64 | 2,2 |
| UDCA | 2,87 | 1,4 | 0,6 |
| muCA | 113,4 | 62,0 | 25,0 |

**Supplementary Table S4J.** Bile acid concentration in Human

| Bile acid | Concentration ${}^{\dagger}$ | SD${}^{\dagger}$ | Relative abundance (%) |
| --- | --- | --- | --- |
| 3,12-DIOXO DCA | 64 | 28 | 6 |
| 3-OXO UDCA | 3.6 | 1.3 | <1 |
| 12B, 3-OXO DCA | 12 | 4 | 1 |
| 12-OXO DCA | 163 | 78 | 16 |
| 3-OXO DCA | 53 | 21 | 5 |
| 3-OXO LCA | 59 | 23 | 6 |
|  |  |  |  |
| CA | 3.2 | 1.4 | <1 |
| DCA | 467 | 215 | 46 |
| LCA | 103 | 38 | 10 |
| isoLCA | 88 | 42 | 9 |

**Supplementary Table S5.** Bacteria involve in bile acid metabolism and bacteria detected in animal samples.

| Bacteria involved in BA metabolis12345 | Bacteria detected in the animal samples |
| --- | --- |
| Bacteroides spp. (B. fragilis, B. thetaiotaomicron) | BACTEROIDES |
| Bacteroides thetaiotaomicron |  |
| Bacteroides spp. (B. distasonis, B. eggerthii, B. vulgatus) |  |
| Bacteroides fragilis |  |
| Bacteroides fragilis + Bacteroides uniformis |  |
| Bacteroides spp. |  |
| Bacteroides pectinophilus WP_008116638.1 |  |
| Bacteroides pectinophilus CAG: 437 CDD 56334.1 |  |
|  |  |
| Eubacterium aerofaciens | EUBACTERIUM COPROSTANOLIGENES GROUP |
| Eubacterium lentum |  |
| Eubacterium spp. V.P.I. 12708 |  |
| Eubacterium sp. CAG:156 CDA29878.1 |  |
| Eubacterium sp. CAG:191 CDB 13476.1 |  |
| Eubacterium sp. CAG:38 CDE37446.1 |  |
| Eubacterium sp. CAG:76 CDF09020.1 |  |
| Eubacterium plexicaudatum WP_004078534567.1 |  |
| Eubacterium ramulus WP_021740004.1 |  |
| Eubacterium hallii WP_022170662.1 |  |
| Eubacterium sp. CAG:161 CCY70318.1 |  |
| Eubacterium ventriosum WP_005360520.1 |  |
| Eubacterium limosum WP_058693606.1 |  |
|  |  |
| Lactobacillus plantarum/Lactobacillus salivarius | LACTOBACILLUS |
| LActobacillus panis WP_047768617.1 |  |
| LActobacillus pontis WP_057806522.1 |  |
| LActobacillus oris WP_003715379.1 |  |
| LActobacillus antri WP_007124818.1 |  |
| LActobacillus oris AMS07483.1 |  |
| LActobacillus oris WP_003713603.1 |  |
| LActobacillus rogosae SFE25013.1 |  |
|  |  |
| Ruminococcus gnavus N53 | RUMINOCOCCUS 1  RUMINOCOCCUS 2  RUMINOCOCCUS TORQUES GROUP  RUMINOCOCCUS GNAVUS GROUP |
| Ruminococcus productus b-52 |  |
| Ruminococcus P01-3 |  |
| Ruminococcus sp. CAG: 403 CDE33478.1 |  |
| Ruminococcus callidus WP_021683261.1 |  |
| Ruminococcus sp. CAG: 254 CCZ84658.1 |  |
| Ruminococcus sp. CAG 330 CDE13720.1 |  |
| Ruminococcus sp. CAG:9-related_41_34_ OLA73075.1 |  |
| Ruminococcus bromii L2-63 CBL14717.1 |  |
|  |  |
| Collinsella aerofaciens ATCC 25986^4^ | COLLINSELLA |
| Collinsella sp. Merseille-P32967 WP_075844024.1 |  |
| Collinsella sp. MS5 WP_002387678.1 |  |
| Collinsella aerofaciens WP_006235414.1 |  |
| Collinsella sp. 4_8_47FAA WP 035137314.1 |  |
| Collinsella stercoris WP_040360544.1 |  |
| Collinsella stercoris DSM 13279 EEA91072.1 |  |
| Collinsella tanakaei WP_009141301.1 |  |
|  |  |
| Faecalinbacterium prausnitzii WP_04493736.1 | FAECALIBACTERIUM |
| Faecalinbacterium prausnitzii cf. KLE1255 EFQ06856.1 |  |
| Faecalinbacterium prausnitzii L2-6 CBK99423.1 |  |
| Faecalinbacterium prausnitzii OLA26681.1 |  |
| Faecalinbacterium sp. CAG:74 CDE48755.1 |  |
|  |  |
| Roseburia hominis CUP92696.1 | ROSEBURIA |
| Roseburia sp. CAG: 10041_57 OLA58684.1 |  |
| Roseburia sp. CAG: 100 CDF46034.1 |  |
| Roseburia sp. CAG: 303 CDE54097.1 |  |

(**1** Bile salt biotransformations by human intestinal bacteria; Jason M. Ridlon, Dae-Joong Kang, and Phillip B. Hylemon, **2** Biotransformations: on steroid nucleus of bile acids; Olga Bortolini, Alessandro Medici, and Silvia Poli, **3** Contribution of the 7 -hydroxysteroid dehydrogenase from Ruminococcus gnavus N53 to ursodeoxycholic acid formation in the human colon; Ja-Young Lee , Hisashi Arai , Yusuke Nakamura , Satoru Fukiya , Masaru Wada , and Atsushi Yokota, **4** Metabolism of Oxo-Bile Acids and Characterization of Recombinant 12α-Hydroxysteroid Dehydrogenases from Bile Acid 7αDehydroxylating Human Gut Bacteria; Heidi Doden,a,b Lina A. Sallam,c Saravanan Devendran,a,b Lindsey Ly,a,d Greta Doden,b Steven L. Daniel,c João M. P. Alves,e Jason M. Ridlona)
